# Manganese-catalyzed oxidation of furfuryl alcohols and furfurals to efficient synthesis of furoic acids†

**DOI:** 10.1039/d4ra05903d

**Published:** 2024-08-27

**Authors:** Tianshu Kou, Weihua Chen, Aimin He, Xiaoru Wang, Xin Li, Bing Cui, Zhiyong Wu, Mingqin Zhao, Min Xie, Zhihui Shao

**Affiliations:** a Technology Center of China Tobacco Hebei Industrial Co., Ltd Shijiazhuang 050051 China; b Flavors and Fragrance Engineering & Technology Research Center of Henan Province, College of Tobacco Science Henan Agricultural University Zhengzhou 450002 China shaozh21@henau.edu.cn

## Abstract

Herein, the direct oxidation of furfuryl alcohols and furfurals to the corresponding furoic acids is performed highly efficiently with potassium hydroxide as the base in the presence of a catalytic amount of PNP pincer manganese catalyst in dioxane. The manganese catalytic system can not only achieve the dehydrogenation conversion of furfuryl alcohols to prepare furoic acids but can also achieve the synthesis of furoic acids from furfurals under more moderate conditions and with less reaction time. In addition, the bifunctional furfuryl alcohols or furfurals can also be efficiently converted into dicarboxylic acid products under optimal reaction conditions.

## Abstract 2

Herein, the direct oxidation of furfuryl alcohols and furfurals to the corresponding furoic acids is performed highly efficiently with potassium hydroxide as the base in the presence of a catalytic amount of PNP pincer manganese catalyst in dioxane.

## Graphical abstract

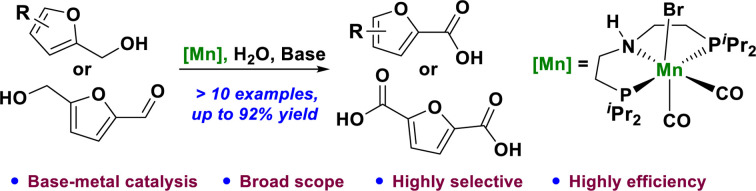

The Maillard reaction is a type of non-enzymatic browning reaction^1^ that widely occurs in baked foods and significantly influences the color, flavor, and other qualities of food. Maillard reaction products can give food a unique aroma and attractive color, but some components, such as 5-hydroxymethylfurfural (5-HMF), are potentially harmful.^2^ 5-HMF is a vital biomass furan compound mainly prepared *via* cellulose hydrolysis and its derivatives. It can be converted into high-value-added fine chemicals through selective oxidation reactions, such as 2,5-furandialdehyde (DFF), 5-hydroxymethyl-2-furan formic acid (HMFCA), 5-formyl-2-furan formic acid (FFCA), and 2,5-furan dicarboxylic acid (FDCA), *etc.*^3^ Among these derivatives, FDCA is considered an ideal alternative to petroleum-derived terephthalic acid as a representative aromatic bio-based chemical platform. Compared to traditional polyethylene terephthalate (PET), polyethylene terephthalate (PEF) prepared using FDCA has significant advantages in terms of sustainability, regeneration, heat resistance, mechanical strength, and gas barrier properties.^4^

Based on the diverse structures and wide sources of alcohols, direct oxidation to produce carboxylic acids has the advantage of using a wide range of substrates and intensive processes. Therefore, the efficient selective oxidation of primary alcohols to carboxylic acids is one of the most critical and challenging issues in organic synthesis and industrial manufacturing and is recognized as a fundamental reaction.^5^ The oxidation of primary alcohols produces aldehydes that may be further oxidized to carboxylic acids. Traditionally, alcohol compounds have been oxidized using stoichiometric oxidants (such as potassium permanganate, chromates, NaClO/NaClO_2_, and oxone) to prepare carboxylic acids.^6^ These processes are often hazardous and produce environmentally harmful stoichiometric amounts of waste, which have little impact on small-scale laboratory synthesis. However, this becomes a severe problem.^7^ With the continuous development of economic globalization, researchers need to explore more efficient chemical conversions to produce valuable products. Research has shown that green and efficient conversion can be achieved by developing highly selective catalysts while improving the yield of the target product and reducing process costs. Therefore, while vigorously developing catalytic technology, exploring new green and environmentally friendly catalysis methods has attracted much attention in recent years.

The synthesis of FDCA from HMF *via* selective catalytic oxidation has been reported previously. These include the use of heterogeneous catalysts,^8^ (photo)electrochemical,^9^ enzymatic,^10^ and biological^11^ oxidation methods (Fig. 1A). However, these catalytic systems require advanced specialized materials and are difficult to improve. Compared to the abovementioned catalytic reaction processes, catalytic homogeneous systems for HMF oxidation are very limited. Transition metal-based complexes have recently been developed for homogeneous catalytic reactions. Reasonable design and selection of appropriate ligands play important roles in fine-tuning the catalytic activity and reaction selectivity of metal complexes. Among them, dehydrogenative coupling reactions have received considerable attention because of their inherently high sub-economy, which can be used as an ideal sustainable approach to obtain carboxylates for the oxidation of alcohols with alkaline water. This is a series of reactions that includes the following processes: alcohol dehydrogenation under the action of a base, coupling of the resulting aldehyde intermediate compound, and alkaline water to form an intermediate, followed by dehydrogenation to obtain carboxylates. The earliest studies on HMF oxidation mainly used Ru^12^ and other noble metal–organic complexes as catalysts (Fig. 1B). The replacement of noble-metal catalysts with economical and environmentally benign earth-abundant metals to catalyst dehydrogenative coupling of HMF with alkaline water to form FDCA has become the research focus in high demand in terms of sustainability.^13^ Encouraged by recent achievements in Mn-catalyzed dehydrogenation reactions,^14^ we developed an Mn-catalyzed general and efficient furfural alcohol and furfural dehydrogenation coupling reaction that afforded a wide range of furanocarboxylic acids (>10 examples) with high selectivity and efficiency (up to 92% yield) using a well-defined pincer manganese catalyst. Furthermore, the reaction using disubstituted furan alcohols or aldehydes as substrates in this catalytic system also yielded furan dicarboxylic acids with high efficiency and selectivity (Fig. 1C).

**Fig. 1:**
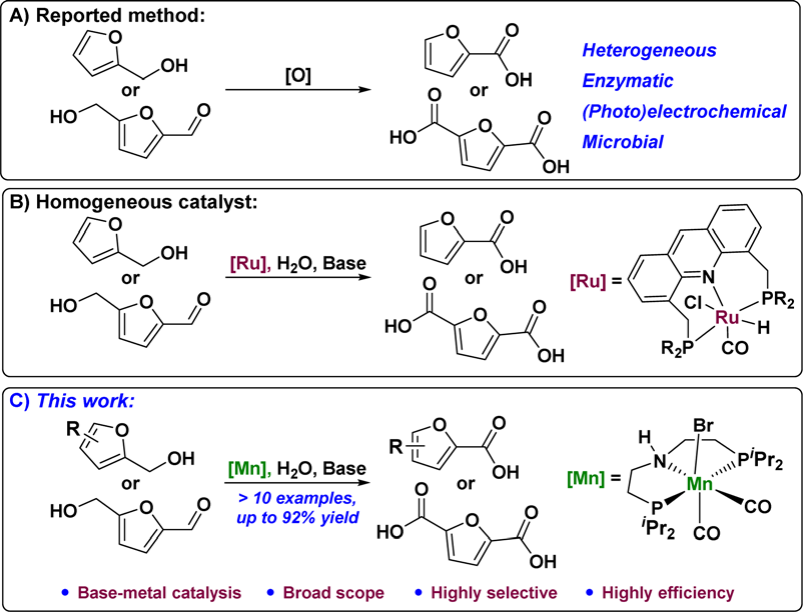
(A) The reported method for the oxidation of furfuryl alcohols to furoic acids. (B) Homogeneous catalytic oxidation of furfuryl alcohols to furoic acid. (C) Mn-catalyzed oxidation of furfuryl alcohol to furoic acid.

Considering the complex molecular structure of HMF, we first studied the catalytic conversion of furfuryl alcohol with a simple structure to achieve the goal of preparing FDCA *via* the catalytic conversion of HMF. Based on our recent research interest in the development of pincer-type manganese catalysts for hydrogenation transfer reactions,^15^ we began our investigations with 1 mmol of furfuryl alcohol (1a) as model reactants and by employing 0.5 mol% [Mn] (mol% based on 1a substrate) as a precatalyst in the present of 1.1 equiv. H_2_O and 1.0 equiv. KOH as the base in 2 mL dioxane at 165 °C for 16 h (Table 1). Initially, the reactivity of a series of well-defined manganese PNP pincer catalysts I–IV in this transformation was tested. The results showed that the four PNP pincer manganese catalysts I–IV afforded good yields (46–92%) and selectivity (Table 1, entries 1–4) for the formation of the desired product 2-furoic acid (2a). Notably, under the same conditions, the more electron-rich ^^i^Pr^PNP-complex, [Mn]-I, gave a much higher yield (92%) for 2a (Table 1, entry 1). However, the Mn catalysts, Mn(CO)_5_Br and MnCl_2,_ displayed very low reactivity in this transformation (Table 1, entries 5 and 6). In addition, we explored the reaction in the absence of a catalyst under the same conditions. The results showed that the target product could not be obtained, further confirming that the PNP pincer ligand had an important impact on the reactivity of the manganese catalyst (Table 1, entry 7). The bases and other reaction parameters were studied in detail after identifying [Mn]-I as the best catalyst for this reaction. For example, using NaOH instead of KOH slightly reduced the yield of 2a (86%) (Table 1, entry 8). Furthermore, the yield of 2a decreased when either stronger (^*t*^BuOK) or weaker (K_2_CO_3_) bases were used (Table 1, entries 9 and 10). After KOH is determined as the best base, the reaction temperature is lowered to 140 °C, and the reaction yield of 2a is also slightly reduced (Table 1, entry 11). Moreover, the target product 2a was obtained in 76% yield by reducing the amount of alkali to 0.8 equivalent (Table 1, entry 13). In contrast, the yield of the target compound was not significantly improved by increasing the amount of alkali to 1.2 equivalent (Table 1, entry 12). Subsequently, we screened the solvents for the reaction, and both toluene and anisole afforded the target product 2a in good yields (85–87%) (Table 1, entry 14 and 15). Finally, we studied the effect of the amount of solvent on the reaction. The results showed that when the amount of dioxane was reduced to 1 mL, the yield of the target product 2a decreased to 85% (Table 1, entry 16). After the optimization of the above conditions, we finally obtained the optimal reaction conditions as follows: 2-furfuryl alcohol (1 mmol), [Mn]-I catalyst (0.5 mol%), KOH (1.0 eq.), H_2_O (1.1 eq.), 2 mL dioxane at 165 °C for 16 h.

**Table 1:** Optimization of reaction conditions^a^

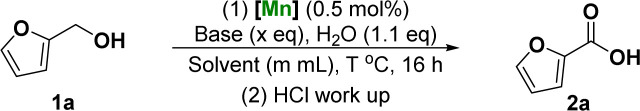							
Entry	[Mn]	Base	*x* [equiv.]	Solvent	*M* [mL]	*T* [°C]	*Y*_2a_ [%]
1	[Mn]-I	KOH	1.0	Dioxane	2	165	92
2	[Mn]-II	KOH	1.0	Dioxane	2	165	87
3	[Mn]-III	KOH	1.0	Dioxane	2	165	81
4	[Mn]-IV	KOH	1.0	Dioxane	2	165	46
5	Mn(CO)_5_Br	KOH	1.0	Dioxane	2	165	<5
6	MnCl_2_	KOH	1.0	Dioxane	2	165	<5
7	None	KOH	1.0	Dioxane	2	165	<5
8	[Mn]-I	KOH	1.0	Dioxane	2	165	86
9	[Mn]-I	^*t*^BuOK	1.0	Dioxane	2	165	27
10	[Mn]-I	K_2_CO_3_	1.0	Dioxane	2	165	14
11	[Mn]-I	KOH	1.0	Dioxane	2	140	83
12	[Mn]-I	KOH	1.2	Dioxane	2	165	92
13	[Mn]-I	KOH	0.8	Dioxane	2	165	76
14	[Mn]-I	KOH	1.0	Toluene	2	165	85
15	[Mn]-I	KOH	1.0	Anisole	2	165	87
16	[Mn]-I	KOH	1.0	Dioxane	1	165	85
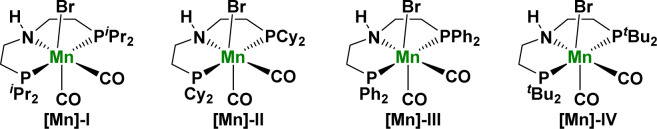							

a Reaction conditions: unless otherwise specified, reactions were performed on a 1 mmol scale of furfuryl alcohol 1a, using 1.0 equiv. of base, 1.1 equiv. of H_2_O, 0.5 mol% of Mn-precatalyst, in 2 mL dioxane at 165 °C for 16 h. Isolated yields are shown.

After determining the optimum conditions for the reaction of furfuryl alcohol to 2-furoic acid catalyzed by [Mn]-I, we further investigated the range of substrates for the reaction process. Scheme 1 summarizes the conversion results of the manganese-catalyzed furoic acid synthesis under optimal reaction conditions. The target compounds were obtained in good to excellent yields using a series of furfuryl alcohols with different substituent structures. Specifically, the corresponding furoic acid products 2a–2b can be obtained in 90–92% yield from furfuryl alcohol at positions C-2 and C-3 of the furfuryl ring. 2-Furfuryl alcohols containing electron-donating groups at the C-5 position, such as methyl and methoxy, reacted smoothly in good-to-excellent yields (84–87%) to obtain the target furoic acid products 2c–2d. Moreover, furfuryl alcohols containing electron-withdrawing substituents, such as chlorine and bromine groups at the C-5 position, can also react to obtain the corresponding products 2e–2g. However, because of the breakage of the carbon–halogen bond under these reductive reaction conditions, the yield was only moderate (72–76%) and lower than that of the products with electron-donating substituents. Furthermore, when the methyl substituent of 3-furfuryl alcohol is in the *ortho*- and the *meta*-site, which can have a larger steric effect, the reaction can also proceed smoothly, and target products with yield of 82–87% can be obtained.

**Scheme 1:**
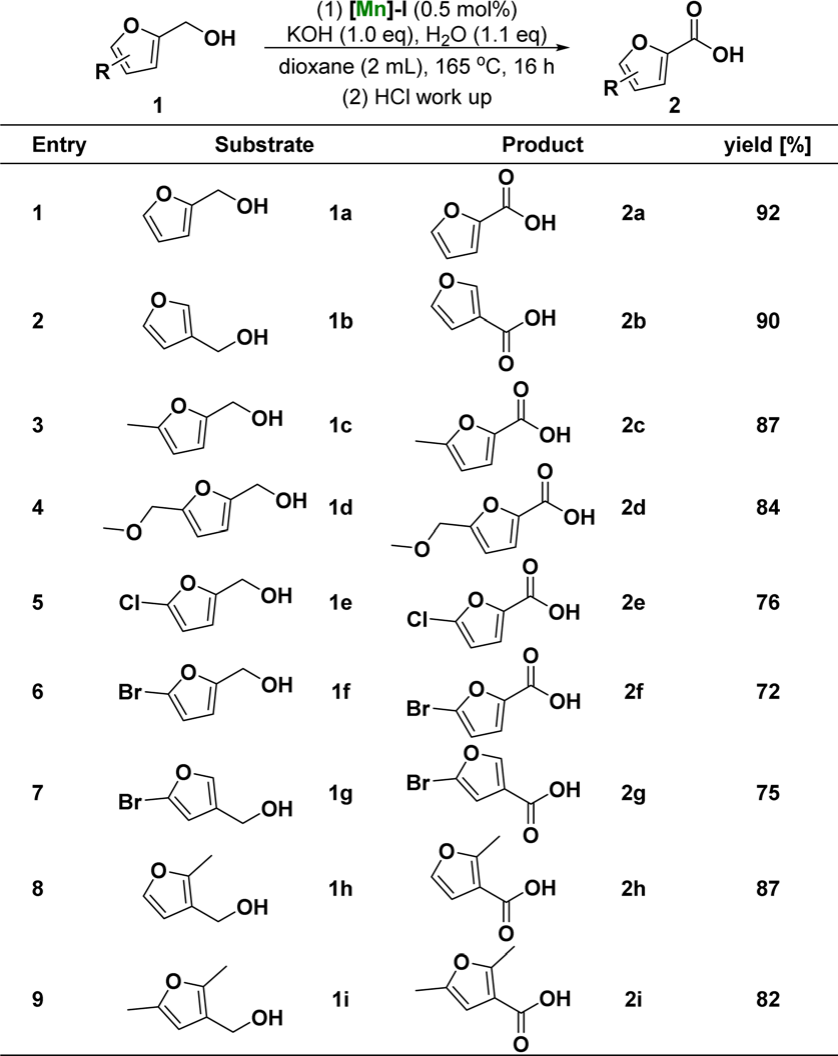
Substrate scope of furfuryl alcohols^*a*^. ^*a*^ Reaction conditions: unless otherwise specified, reactions were performed on a 1 mmol scale of furfuryl alcohols 1, using 1.0 equiv. of KOH, 1.1 equiv. of H_2_O, 0.5 mol% of [Mn]-I, in 2 mL dioxane at 165 °C for 16 h. Isolated yields are shown.

Subsequently, the catalytic system was used to study the reactivity of the furfurals (Scheme 2). Compared with the above optimal reaction conditions, the reaction of [Mn]-I catalyst with furfurals can occur at lower reaction temperature (120 °C *vs.* 165 °C) and shorter reaction time (6 h *vs.* 16 h), and the products of furoic acid can be obtained with excellent yield. During substrate expansion, we studied the reactivity of furfurals with aldehyde substituents at the C-2 and C-3 positions, yielding 2a and 2b in 89% and 87% yields, respectively. Under the same reaction conditions, electron-donating groups, such as 5-methyl, and electron-withdrawing groups, such as 5-bromo substituted furfural, were successfully converted to the corresponding furoic acid products 2c and 2f in 87% and 75% yields, respectively. In this study, the rate of hydroxyl oxidation was much lower than that of aldehyde oxidation. These results illustrated the higher activity of the aldehyde groups.

**Scheme 2:**
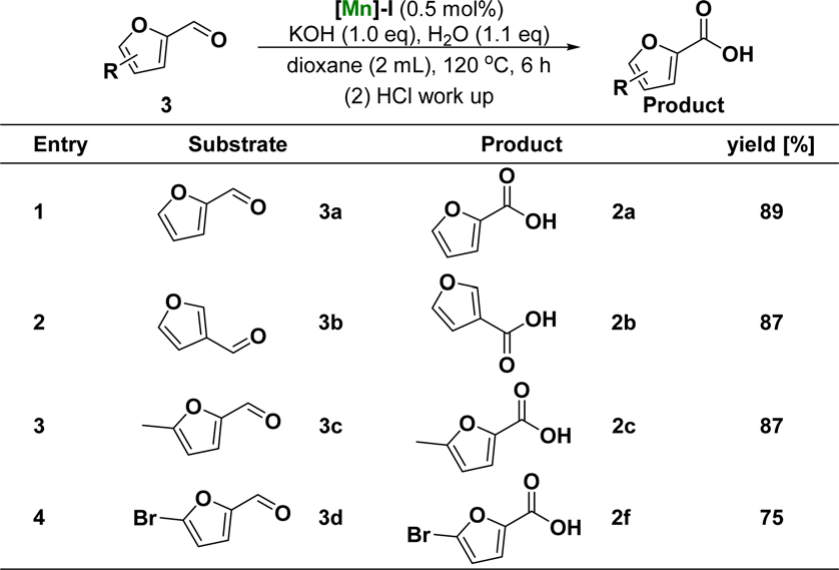
Substrate scope of furfurals^*a*^. ^*a*^Reaction conditions: unless otherwise specified, reactions were performed on a 1 mmol scale of furanaldehyde 3, using 1.0 equiv. of KOH, 1.1 equiv. of H_2_O, 0.5 mol% of [Mn]-I, in 2 mL dioxane at 120 °C for 6 h. Isolated yields are shown.

To further demonstrate the universality of our developed method and realize the oxidation of HMF to FDCA. Difunctionally substituted furyl alcohols and aldehydes were also used in this catalytic reaction system to verify the catalytic activity (4a–4c), and the results are shown in Scheme 3. In contrast to the previous optimal reaction conditions, furan dicarboxylic acid products can be prepared in good yield by adding 2 equivalents of the KOH and 2.2 equivalents of the H_2_O at 1 mmol of the substrate. Specifically, functional groups are located at the C-2 and C-5 positions, such as dihydroxymethyl, bialdehyde, and furan substrates with aldehyde and hydroxymethyl groups. Both functional groups could react to obtain the corresponding furan dicarboxylic acid compound 5a in good yield. In addition, when the dihydroxymethyl functional group was located at the ortho-positions, such as C-2, C-3, and C-3, C-4, dihydroxymethylfuran could also participate in the reaction. The yields of furan dicarboxylic acids 5b and 5c were 67% and 69%, respectively.

**Scheme 3:**
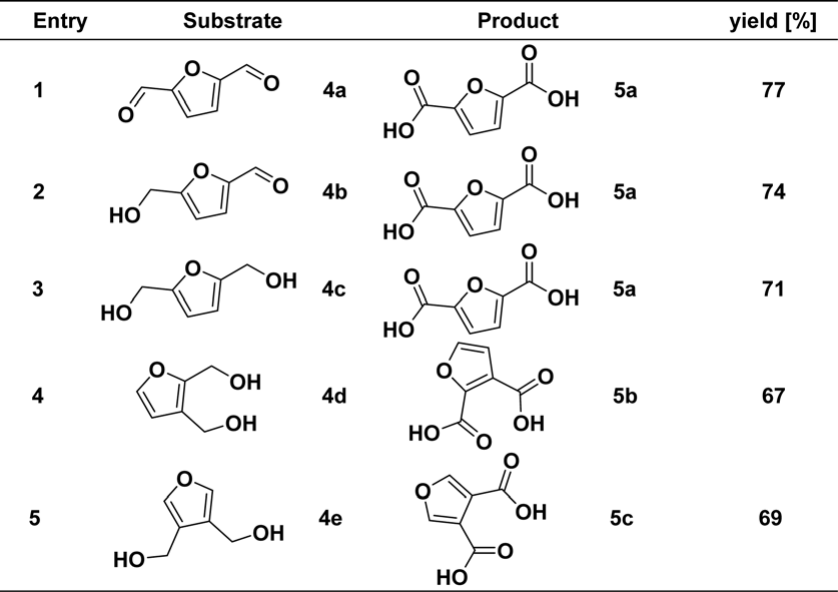
Substrate scope of difunctional substituted furyl alcohols and aldehydes^*a*^. ^*a*^ Reaction conditions: unless otherwise specified, reactions were performed on a 1 mmol scale of 4, using 2.0 equiv. of KOH, 2.2 equiv. of H_2_O, 0.5 mol% of [Mn]-I, in 2 mL dioxane at 165 °C for 16 h. Isolated yields are shown.

To further understand the mechanism of the dehydrogenative transformation of furfuryl alcohol catalyzed by Mn, a series of controlled experiments was designed, as shown in Fig. 2. To investigate whether the reaction underwent furfural or furfuryl carboxylate reaction intermediates, the dehydrogenation of furfuryl alcohol was catalyzed using a catalytic amount of base to obtain 31% furfural 3a and a small amount of furfuryl carboxylate 6 (Fig. 2A). The reaction using furfuryl carboxylate 6 as the starting material was investigated under standard conditions. The results showed that 2-furoic acid 2a was obtained in 82% yield (Fig. 2B). We also attempted the reaction using furfural as the substrate, and 2a obtained a 89% yield under the standard reaction conditions (Fig. 2C). Finally, the reaction of furfural 3a was studied without the addition of [Mn]-I and the results showed that there was no formation of product 2a or its corresponding ester (Fig. 2D). These results suggest that the reaction intermediate may be furfural rather than carboxylate. In other words, the possible reaction mechanism of the manganese-catalyzed dehydrogenation of furfuryl alcohol to furoic acid is that alcohol dehydrogenates to form aldehyde intermediates. The aldehyde intermediates undergo a nucleophilic addition reaction with hydroxide ions and further dehydrogenate to form carboxylic acid products under the action of the catalyst.

**Fig. 2:**
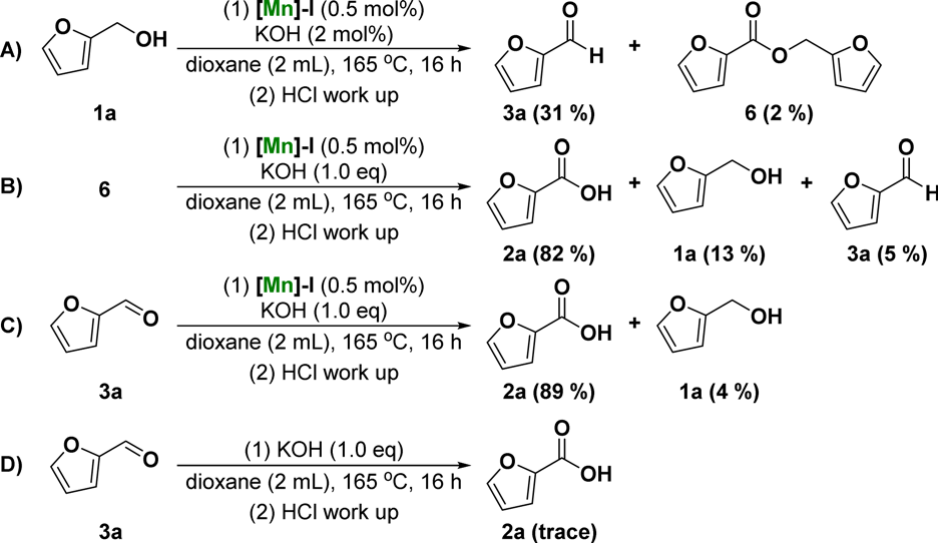
Control experiments.

Based on the above experimental results and the basis of our previous research, we propose a possible reaction mechanism in which Mn catalyzes the dehydrogenation coupling of 2-furfuryl alcohol and hydroxide to produce 2-furoic acid products (Fig. 3). First, the [Mn]-I catalyst removed a hydrogen bromide molecule under the action of KOH to produce the amino–manganese complex [Mn]-IA. Complex [Mn]-IA was further transformed into furfuryl manganese complex [Mn]-IB by binding to the alcohol substrate. Manganese complex [Mn]-IB undergoes a β-hydrogen elimination process to remove a molecule of hydrogen and obtain an intermediate [Mn]-IC, and the intermediate [Mn]-IC undergoes nucleophilic addition reaction with KOH to obtain a manganese complex [Mn]-ID. The [Mn]-ID complex continues to undergo β-hydrogen elimination to release another molecule of hydrogen and reform the amino manganese complex [Mn]-IA, thus obtaining the final carboxylate product and completing the catalytic cycle.

**Fig. 3:**
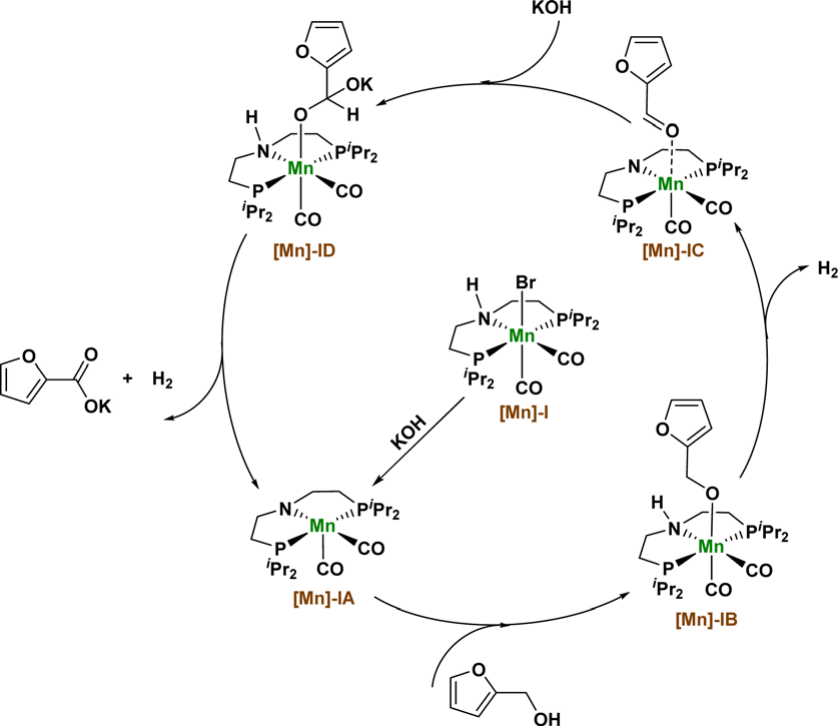
Proposed reaction mechanism.

We developed an effective and sustainable manganese pincer catalyst system for the efficient synthesis of furoic acids *via* the manganese-catalyzed dehydrogenation coupling of furfuryl alcohols and furfurals with hydroxide in the presence of dioxane as the solvent. Reactions with a variety of furfuryl alcohol substrates (>10 examples) proceeded smoothly, with up to 92% product yield and excellent functional group tolerance (tested with a series of reducible functional groups such as carbon–halogen bonds and carbon–oxygen bonds). Moreover, the proposed reaction system is suitable for furfurals. It can react at lower temperatures and shorter reaction times with good yields. Mechanistic studies, including control experiments, were used to identify the key reaction intermediates in the catalytic cycle. In view of the easy availability of the non-noble metal catalyst and biomass-derived substrates used here, this catalytic method is expected to complement current methods for the oxidation of furfuryl alcohols in organic synthesis.

## Data availability

The data supporting this article have been included as part of the ESI.†

## Conflicts of interest

There are no conflicts to declare.

## Supplementary Material

RA-014-D4RA05903D-s001
