# Supplementary material for: Manganese-catalyzed oxidation of furfuryl alcohols and furfurals to efficient synthesis of furoic acids
Source: RSC Adv. 2024 Aug 27;14(37):27060–5. doi: 10.1039/d4ra05903d (PMC11348847; doi:10.1039/d4ra05903d)
Supplement: RA-014-D4RA05903D-s001 [file RA-014-D4RA05903D-s001.pdf]

Supplementary Information (SI) for RSC Advances.
This journal is © The Royal Society of Chemistry 2024

Supporting Information

Manganese-Catalyzed Oxidation of Furfuryl Alcohols and Furfurals to Efficient Synthesis of Furoic Acids

Tianshu Kou,[a] Weihua Chen,[a] Aimin He,[a] Xiaoru Wang,[a] Xin Li,[a] Bing Cui,[b] Zhiyong Wu,[b] Mingqin Zhao,*[b] Min Xie,*[a] and Zhihui Shao*[b]

[a] Technology Center of China Tobacco Hebei Industrial Co., LTD, Shijiazhuang 050051, China

[b] Flavors and Fragrance Engineering & Technology Research Center of Henan Province, College of Tobacco Science, Henan Agricultural University, Zhengzhou 450000, China.

Table of Contents

General Considerations 3

General procedures for the dehydrogenation coupling of 2-furfuryl alcohol with hydroxide to 2-furoic acid catalyzed by [Mn]-I 4

General procedures for the dehydrogenation coupling of furfuryl alcohols with hydroxide to furoic acids catalyzed by [Mn]-I 5

General procedures for the dehydrogenation coupling of furfurals with hydroxide to furoic acids catalyzed by [Mn]-I 6

General procedures for the dehydrogenation coupling of difunctional substituted furyl alcohols and aldehydes with hydroxide to furan dicarboxylic acid catalyzed by [Mn]-I7

Characterization Data of Products..8

Reference..11

General Considerations

The synthesis of ligands and preparation of catalysts in this work were reported in our previous publication[1]. Air and moisture sensitive reactions were carried out in glovebox or in over-dried glassware sealed with rubber septa using standard schlenk techniques. Most solvents used were dried over solvent purification system (Innovative Technology PS-MD-5) and alcohol solvents were dried over calcium hydride. Deuterated solvents were purchased from Cambridge Isotope Laboratories, vented and distilled over calcium hydride. All chemicals were purchased from commercial sources with purity over 95% and used without further purification. NMR spectra were received using a Bruker 400 MHz spectrometer. Chemical shifts are reported in ppm relative to the deuterated solvent. GC analysis were carried out on SHIMADAZU GC 2010 PLUS system. (Column: SH-Rtx-200, 30 m x 0.25 mm x 0.25 μm). GC/MS analyses were carried out on an GC-MS-QP2010 SE W system equipped with aSH-Rxi-5Sil MS 30 meter, 0.25 mmID, 0.25 um df. High resolution exact mass measurements (HRMS) were performed on Thermo SCIENTIFIC Q EXACTIVE.

General procedures for the dehydrogenation coupling of 2-furfuryl alcohol with hydroxide to 2-furoic acid catalyzed by [Mn]-I

Under the protection of argon, 2-furfuryl alcohol 1 (1 mmol), **[Mn]** (0.005 mmol, 0.5 mol%), Base (1.0 mmol, 1.0 equiv), H_2O (20 mg, 1.1 mmol, 1.1 equiv), and 1,4-dioxane (2 mL) were added sequentially to a 25 mL schlenck tube equipped with a magnetic stir bar. The reaction was stirred at 165 °C for 16 hours. After cooling to room temperature, water (5 mL) was added and the mixture was extracted with diethyl ether (3 × 5 mL). Then the aqueous phase was acidified with 6M HCl and extracted with ethyl acetate (3 × 10 mL). The combined ethyl acetate solution were washed with brine (15 mL), dried over anhydrous Na_2SO_4, and evaporated to dryness under reduced pressure, the pure acids was obtained and weighted for calculating the yield. Yield = n (acid) / n (alcohol).

Table S1. Optimization of Reaction Conditions.

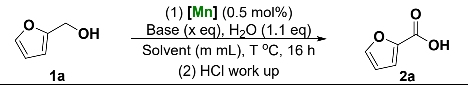

Entry	[Mn]	Base	x [equiv.]	Solvent	m [mL]	T [°C]	Y_{2a} [%]
1	[Mn]-I	KOH	1.0	Dioxane	2	165	92
2	[Mn]-II	KOH	1.0	Dioxane	2	165	87
3	[Mn]-III	KOH	1.0	Dioxane	2	165	81
4	[Mn]-IV	KOH	1.0	Dioxane	2	165	46
5	$Mn(CO)_5Br$	KOH	1.0	Dioxane	2	165	< 5
6	$MnCl_2$	KOH	1.0	Dioxane	2	165	< 5
7	none	KOH	1.0	Dioxane	2	165	< 5
8	[Mn]-I	NaOH	1.0	Dioxane	2	165	86
9	[Mn]-I	^tBuOK	1.0	Dioxane	2	165	27
10	[Mn]-I	K_2CO_3	1.0	Dioxane	2	165	14
11	[Mn]-I	KOH	1.0	Dioxane	2	140	83
12	[Mn]-I	KOH	1.2	Dioxane	2	165	92
13	[Mn]-I	KOH	0.8	Dioxane	2	165	76
14	[Mn]-I	KOH	1.0	Toluene	2	165	85
15	[Mn]-I	KOH	1.0	Anisole	2	165	87
16	[Mn]-I	KOH	1.0	Dioxane	1	165	85

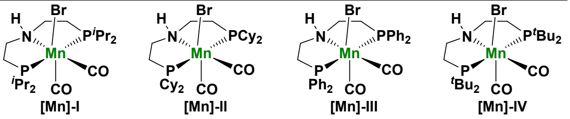

[a] Reaction conditions: Unless otherwise specified, reactions were performed on a 1 mmol scale of furfuryl alcohol **1a**, using 1.0 equiv of base, 1.1 equiv of H_2O, 0.5 mol% of **Mn**-precatalyst, in 2 mL dioxane at 165 °C for 16 h. Isolated yields are shown.

General procedures for the dehydrogenation coupling of furfuryl alcohols with hydroxide to furoic acids catalyzed by [Mn]-I

Under the protection of argon, furfuryl alcohols **1** (1 mmol), **[Mn]-I** (2.5 mg, 0.005 mmol, 0.5 mol%), KOH (56 mg, 1.0 mmol, 1.0 equiv), H_2O (20 mg, 1.1 mmol, 1.1 equiv), and 1,4-dioxane (2 mL) were added sequentially to a 25 mL schlenck tube equipped with a magnetic stir bar. The reaction was stirred at 165 ^{o}C for 16 hours. After cooling to room temperature, water (5 mL) was added and the mixture was extracted with diethyl ether (3 × 5 mL). Then the aqueous phase was acidified with 6M HCl and extracted with ethyl acetate (3 × 10 mL). The combined ethyl acetate solution were washed with brine (15 mL), dried over anhydrous Na_2SO_4, and evaporated to dryness under reduced pressure, the pure acids was obtained and weighted for calculating the yield. Yield = n (acid) / n (alcohol).

General procedures for the dehydrogenation coupling of furfurals with hydroxide to furoic acids catalyzed by [Mn]-I

Under the protection of argon, furfurals **3** (1 mmol), **[Mn]-I** (2.5 mg, 0.005 mmol, 0.5 mol%), KOH (56 mg, 1.0 mmol, 1.0 equiv), H_2O (20 mg, 1.1 mmol, 1.1 equiv), and 1,4-dioxane (2 mL) were added sequentially to a 25 mL schlenck tube equipped with a magnetic stir bar. The reaction was stirred at 120 ^{o}C for 6 hours. After cooling to room temperature, water (5 mL) was added and the mixture was extracted with diethyl ether (3 × 5 mL). Then the aqueous phase was acidified with 6M HCl and extracted with ethyl acetate (3 × 10 mL). The combined ethyl acetate solution were washed with brine (15 mL), dried over anhydrous Na_2SO_4, and evaporated to dryness under reduced pressure, the pure acids was obtained and weighted for calculating the yield. Yield = n (acid) / n (aldehyde).

General procedures for the dehydrogenation coupling of difunctional substituted furyl alcohols and aldehydes with hydroxide to furan dicarboxylic acid catalyzed by [Mn]-I

Under the protection of argon, substrate **4** (1 mmol), **[Mn]-I** (2.5 mg, 0.005 mmol, 0.5 mol%), KOH (112 mg, 2.0 mmol, 2.0 equiv), H_2O (40 mg, 2.2 mmol, 2.2 equiv), and 1,4-dioxane (2 mL) were added sequentially to a 25 mL schlenck tube equipped with a magnetic stir bar. The reaction was stirred at 165 ^{o}C for 16 hours. After cooling to room temperature, water (5 mL) was added and the mixture was extracted with diethyl ether (3 × 5 mL). Then the aqueous phase was acidified with 6M HCl and extracted with ethyl acetate (3 × 10 mL). The combined ethyl acetate solution were washed with brine (15 mL), dried over anhydrous Na_2SO_4, and evaporated to dryness under reduced pressure, the pure acids was obtained and weighted for calculating the yield. Yield = n (acid) / n (substrate).

Characterization Data of Products

2-furoic acid **2a**[2]

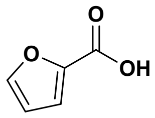

Product was isolated as white solid. ^{1}H NMR (400 MHz, DMSO-d_6) δ 13.06 (s, 1H), 8.09 – 7.67 (m, 1H), 7.22 (dd, J = 3.5, 0.9 Hz, 1H), 6.65 (dd, J = 3.5, 1.8 Hz, 1H). ^{13}C NMR (101 MHz, DMSO-d_6) δ 159.77 (s), 147.47 (s), 145.36 (s), 118.15 (s), 112.53 (s).

3-furoic acid **2b**[3]

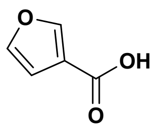

Product was isolated as white solid. ^{1}H NMR (400 MHz, DMSO-d_6) δ 12.54 (s, 1H), 8.29 (s, 1H), 7.77 (s, 1H), 6.73 (d, *J* = 1.9 Hz, 1H). ^{13}C NMR (101 MHz, DMSO-d_6) δ 164.34 (s), 148.56 (s), 145.04 (s), 120.28 (s), 110.31 (s).

5-Methyl-2-furoic acid **2c**[4]

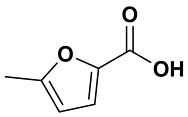

Product was isolated as white solid. ^{1}H NMR (400 MHz, DMSO-d_6) δ 7.10 (d, *J* = 3.4 Hz, 1H), 6.28 (d, *J* = 3.2 Hz, 1H), 2.33 (s, 3H). ^{13}C NMR (101 MHz, DMSO-d_6) δ 159.70 (s), 157.01 (s), 143.74 (s), 119.56 (s), 109.05 (s), 13.94 (s).

5-(methoxymethyl)-2-furoic acid **2d**[5]

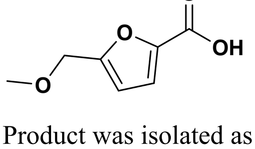

Product was isolated as white solid. ^{1}H NMR (400 MHz, DMSO-d_6) δ 7.17 (d, *J* = 3.4 Hz, 1H), 6.60 (d, *J* = 3.4 Hz, 1H), 4.40 (s, 2H), 3.27 (s, 3H). ^{13}C NMR (101 MHz, DMSO-d_6) δ 159.71 (s), 156.04 (s), 145.12 (s), 118.78 (s), 111.81 (s), 65.85 (s), 57.92 (s).

5-chloro-2-furoic acid **2e**[6]

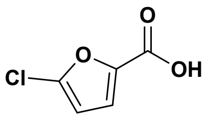

Product was isolated as white solid. ^{1}H NMR (400 MHz, DMSO-d_6) δ 7.29 (d, J = 3.5 Hz, 1H), 6.69 (d, J = 3.6 Hz, 1H). ^{13}C NMR (101 MHz, DMSO-d_6) δ 158.71 (s), 144.97 (s), 139.57 (s), 120.45 (s), 110.04 (s).

5-bromofuroic acid **2f**[7]

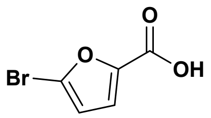

Product was isolated as white solid. ^{1}H NMR (400 MHz, DMSO-d_6) δ 7.25 (d, J = 3.6 Hz, 1H), 6.80 (d, J = 3.6 Hz, 1H). ^{13}C NMR (101 MHz, DMSO-d_6) δ 158.69 (s), 147.25 (s), 127.18 (s), 120.52 (s), 114.82 (s).

2-bromofuran-4-carboxylic acid **2g**[8]

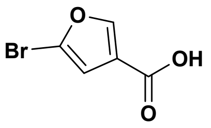

Product was isolated as white solid. ^{1}H NMR (400 MHz, DMSO-d_6) δ 8.37 (s, 1H), 6.84 (s, 1H). ^{13}C NMR (101 MHz, DMSO-d_6) δ 163.19 (s), 150.37 (s), 123.96 (s), 122.66 (s), 111.93 (s).

2-methyl-3-furoic acid **2h**[9]

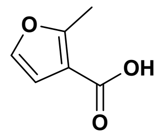

Product was isolated as white solid. ^{1}H NMR (400 MHz, DMSO-d_6) δ 7.55 (d, J = 2.0 Hz, 1H), 6.62 (d, J = 1.9 Hz, 1H), 2.51 (s, 3H). ^{13}C NMR (101 MHz, DMSO-d_6) δ 165.17 (s), 158.69 (s), 141.64 (s), 114.21 (s), 111.28 (s), 13.76 (s).

2,5-dimethyl-3-furoic acid **2i**[10]

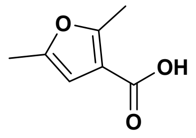

Product was isolated as white solid. ^{1}H NMR (400 MHz, DMSO-d_6) δ 6.21 (d, J = 1.3 Hz, 1H), 2.45 (s, 3H), 2.21 (s, 3H). ^{13}C NMR (101 MHz, DMSO-d_6) δ 165.20 (s), 157.18 (s), 150.15 (s), 114.58 (s), 106.96 (s), 13.70 (s), 13.28 (s).

2,5-furandicarboxylic acid **5a**[4]

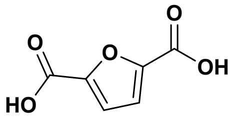

Product was isolated as white solid. ^{1}H NMR (400 MHz, DMSO-d_6) δ 7.29 (s, 2H). ^{13}C NMR (101 MHz, DMSO-d_6) δ 159.35 (s), 147.47 (s), 118.84 (s).

2,3-furandicarboxylic acid **5b**[11]

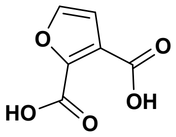

Product was isolated as white solid. ^{1}H NMR (400 MHz, DMSO-d_6) δ 7.93 (d, J = 1.8 Hz, 1H), 6.90 (d, J = 1.8 Hz, 1H). ^{13}C NMR (101 MHz, DMSO-d_6) δ 165.09 (s), 159.78 (s), 146.21 (s), 145.54 (s), 124.81 (s), 113.44 (s).

3,4-furandicarboxylic acid **5c**[12]

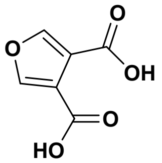

Product was isolated as white solid. ^{1}H NMR (400 MHz, DMSO-d_6) δ 8.41 (s, 2H). ^{13}C NMR (101 MHz, DMSO-d_6) δ 164.51 (s), 150.94 (s), 118.58 (s).

Reference

[1] S. Fu, Z. Shao, Y. Wang, Q. Liu, *J. Am. Chem. Soc.* **2017**, *139*, 11941-11948.

[2] H. S. Yang, L. Macha, H.-J. Ha, J. W. Yang, *Org. Chem. Front.* **2021**, *8*, 53-60.

[3] S. L. Yedage, B. M. Bhanage, *J. Org. Chem.* **2017**, *82*, 5769-5781.

[4] K. Gupta, R. K. Rai, A. D. Dwivedi, S. K. Singh, *ChemCatChem* **2017**, *9*, 2760-2767.

[5] J.-T. Sha, J. Yu, J.-R. Dai, G.-H. Lu, M.-H. Zong, N. Li, *Adv. Synth. Catal.* **2024**, *366*, 1189-1198.

[6] D. A. Sandham, L. Barker, D. Beattie, D. Beer, L. Bidlake, D. Bentley, K. D. Butler, S. Craig, D. Farr, C. Ffoulkes-Jones, J. R. Fozard, S. Haberthuer, C. Howes, D. Hynx, S. Jeffers, T. H. Keller, P. A. Kirkham, J. C. Maas, L. Mazzoni, A. Nicholls, G. E. Pilgrim, E. Schaebulin, G. M. Spooner, R. Stringer, P. Tranter, K. L. Turner, M. F. Tweed, C. Walker, S. J. Watson, B. M. Cuenoud, *Bioorgan. Med. Chem.* **2004**, *12*, 5213-5224.

[7] Z. Chen, H. Zhou, F. Kong, M. Wang, *Appl. Catal. B Environ.* **2022**, *309*, 121281.

[8] T. Terai, M. Kohno, G. Boncompain, S. Sugiyama, N. Saito, R. Fujikake, T. Ueno, T. Komatsu, K. Hanaoka, T. Okabe, Y. Urano, F. Perez, T. Nagano, *J. Am. Chem. Soc.* **2015**, *137*, 10464-10467.

[9] W. S. Trahanovsky, Y. J. Huang, M. Leung, *J. Org. Chem.* **1994**, *59*, 2594-2598.

[10] I. H. Eissa, H. Mohammad, O. A. Qassem, W. Younis, T. M. Abdelghany, A. Elshafeey, M. M. Abd Rabo Moustafa, M. N. Seleem, A. S. Mayhoub, *Eur. J. Med. Chem.* **2017**, *130*, 73-85.

[11] K. Miller-Moslin, S. Peukert, R. K. Jain, M. A. McEwan, R. Karki, L. Llamas, N. Yusuff, F. He, Y. Li, Y. Sun, M. Dai, L. Perez, W. Michael, T. Sheng, H. Lei, R. Zhang, J. Williams, A. Bourret, A. Ramamurthy, J. Yuan, R. Guo, M. Matsumoto, A. Vattay, W. Maniara, A. Amaral, M. Dorsch, J. F. Kelleher, III, *J. Med. Chem.* **2009**, *52*, 3954-3968.

[12] T. Ikeda, H. Ikeda, Y. Takahashi, M. Yamada, K. Mizuno, S. Tero-Kubota, S. Yamauchi, *J. Am. Chem. Soc.* **2008**, *130*, 2466-2472.

Characterization Data of Products

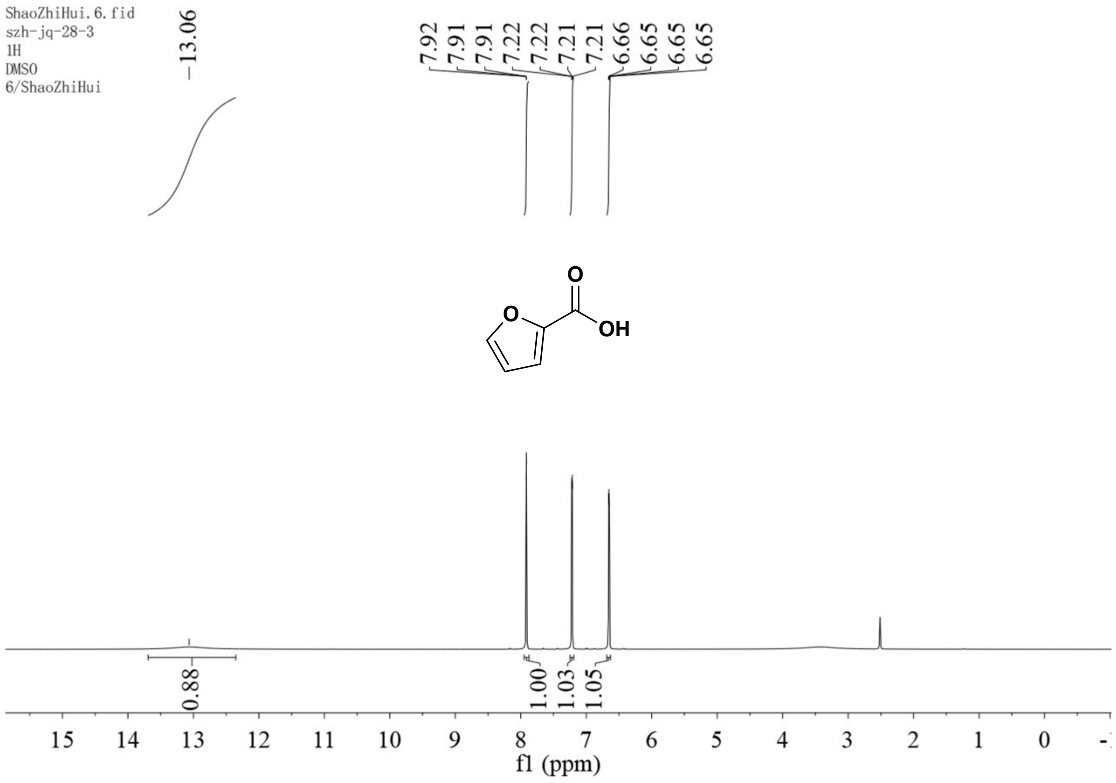

^{1}H NMR-spectrum (400 MHz, DMSO-d_6) of **2a**

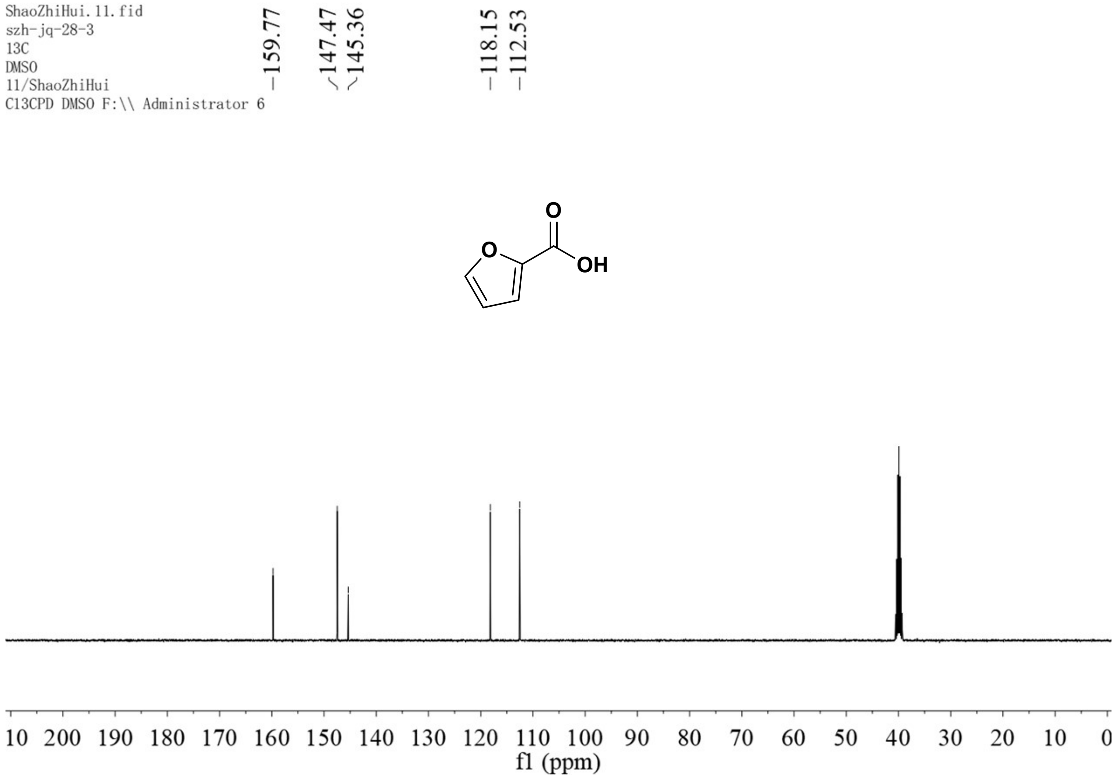

^{13}C NMR-spectrum (101 MHz, DMSO-d_6) of **2a**

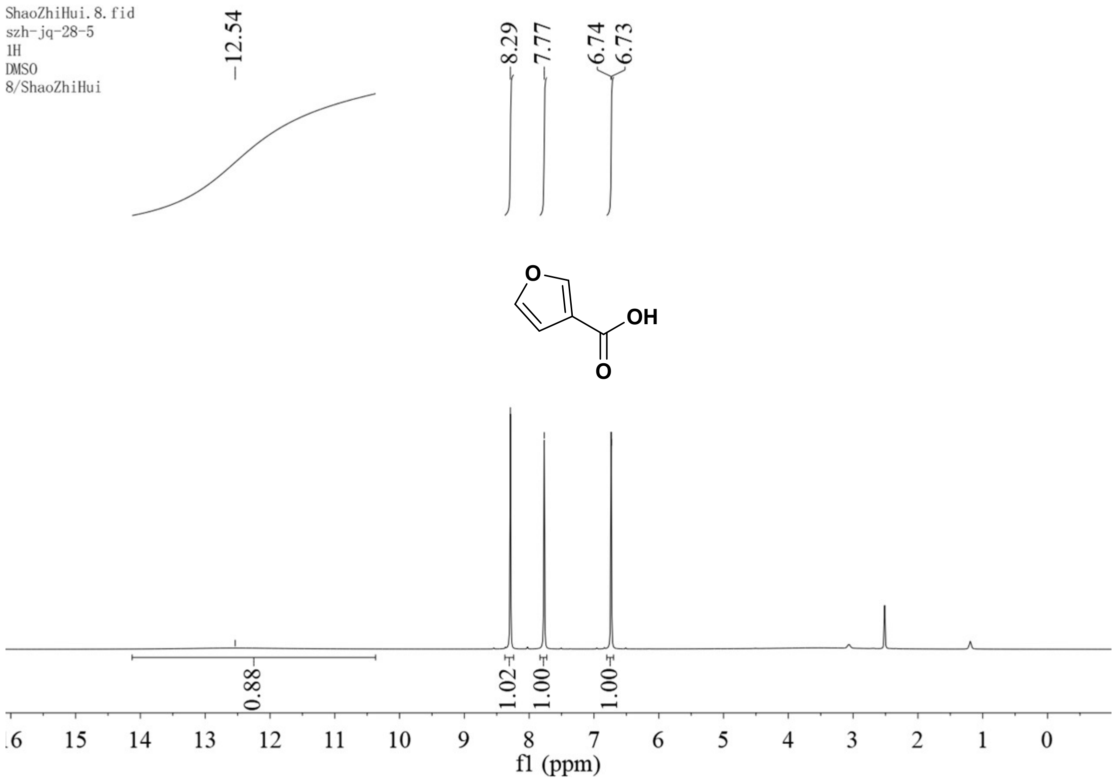

^{1}H NMR-spectrum (400 MHz, DMSO-d_6) of **2b**

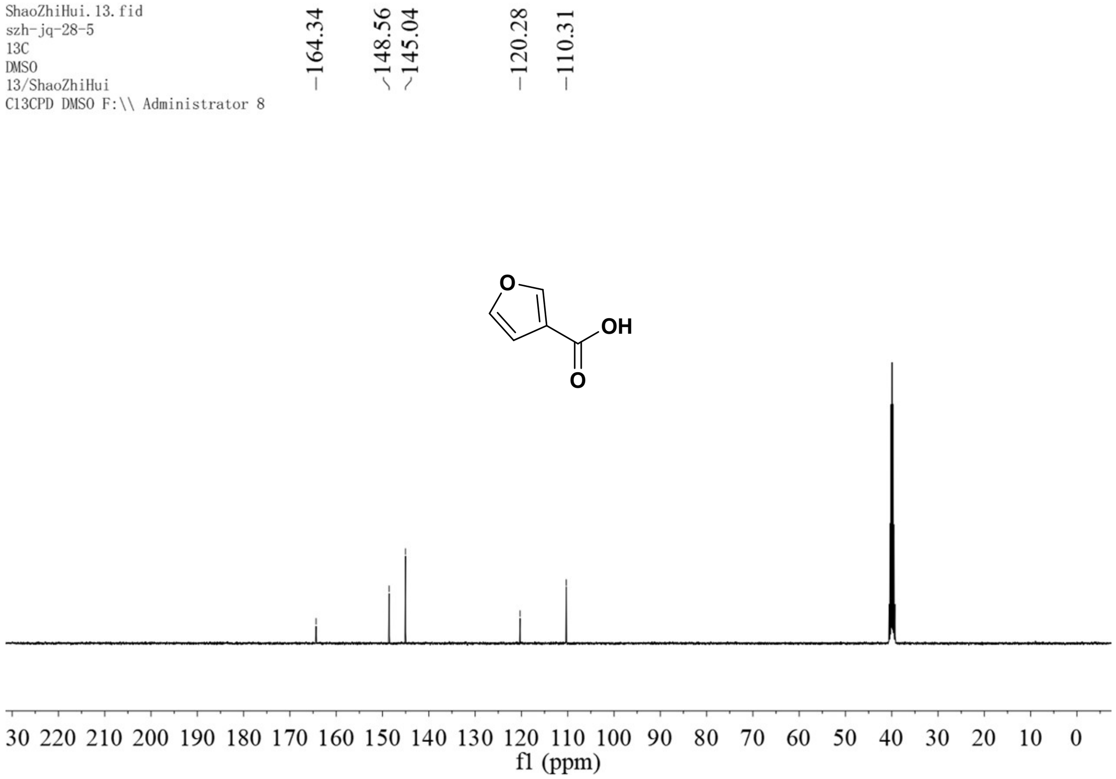

^{13}C NMR-spectrum (101 MHz, DMSO-d_6) of **2b**

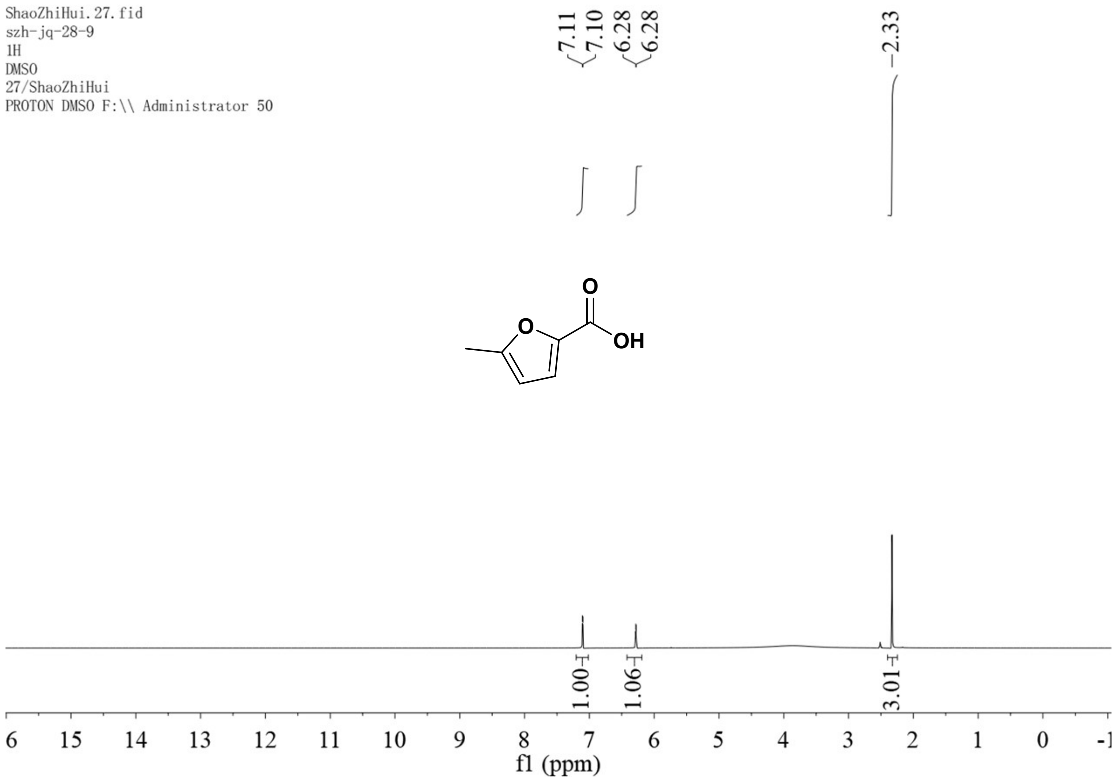

^{1}H NMR-spectrum (400 MHz, DMSO-d_6) of **2c**

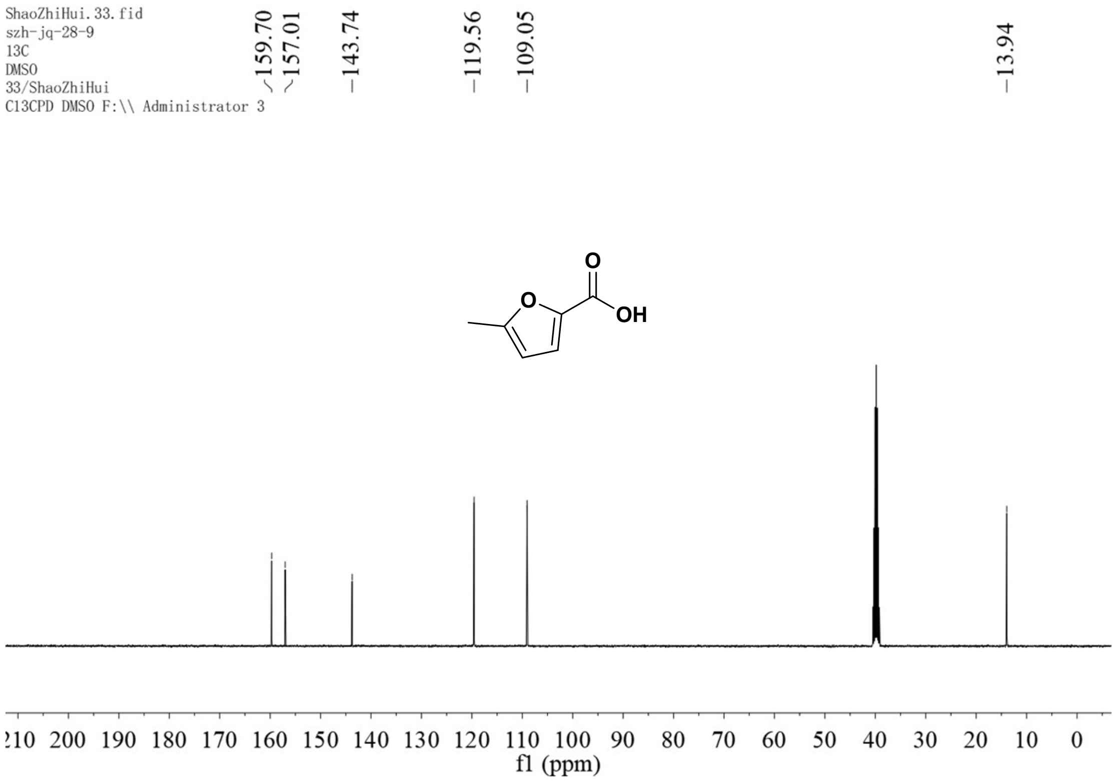

^{13}C NMR-spectrum (101 MHz, DMSO-d_6) of **2c**

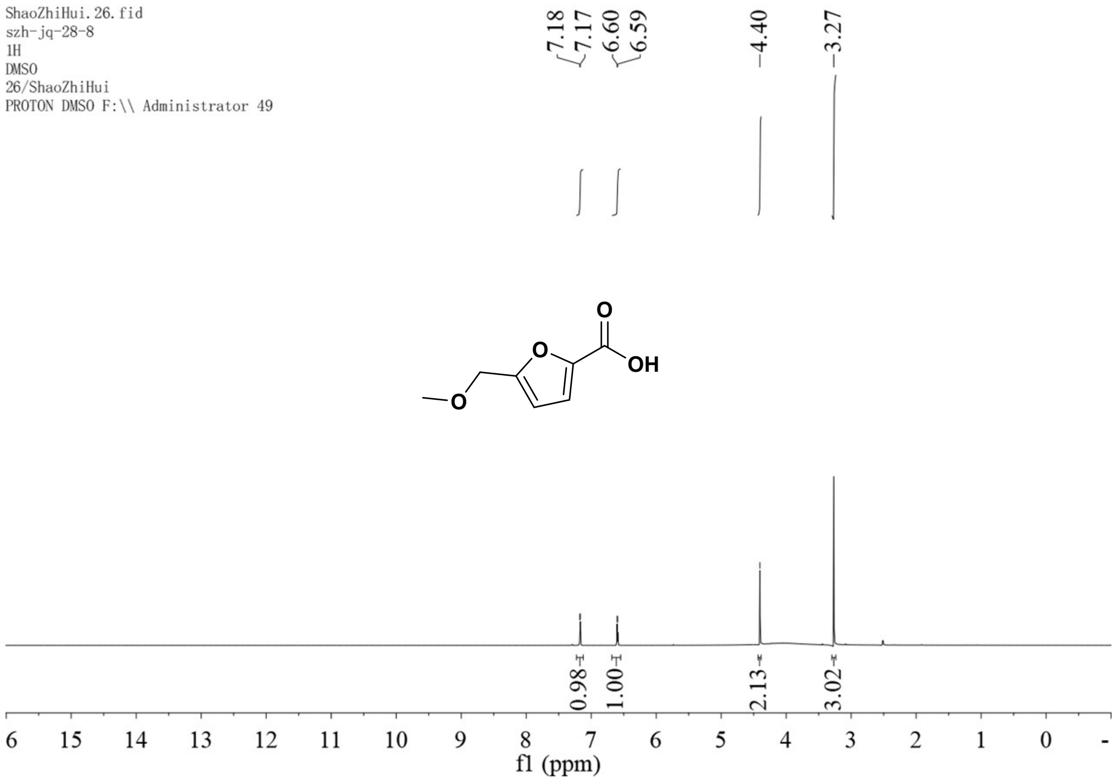

^{1}H NMR-spectrum (400 MHz, DMSO-*d6*) of **2d**

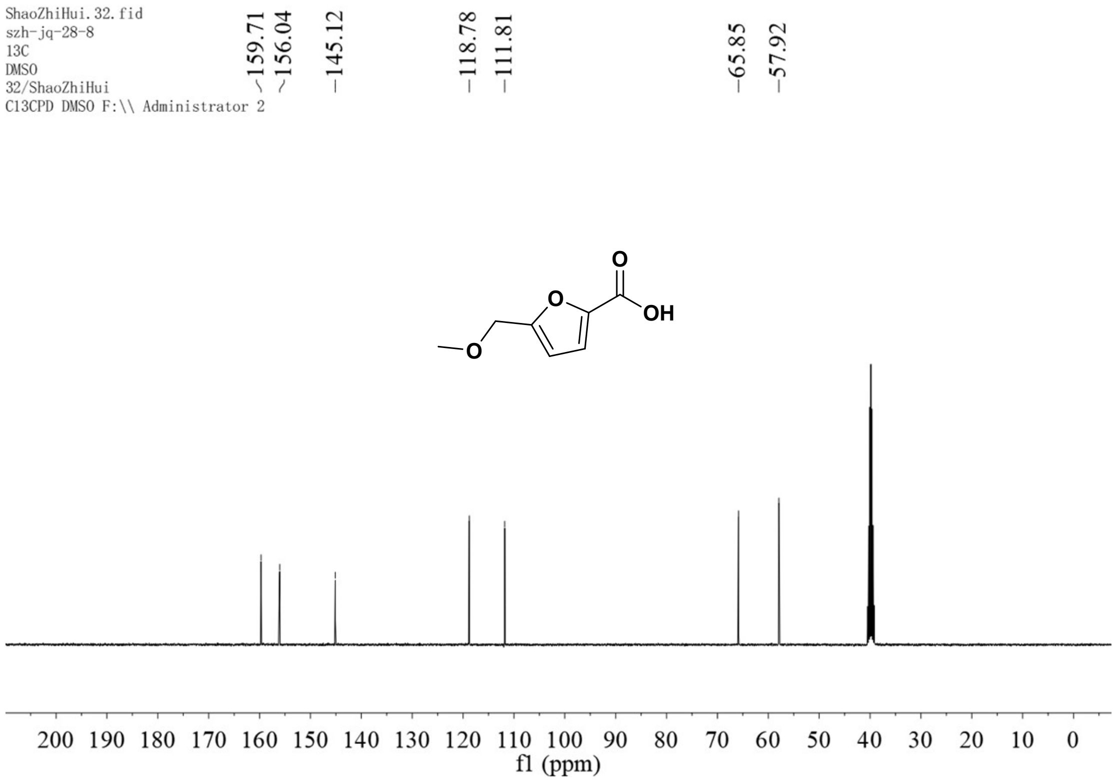

^{13}C NMR-spectrum (101 MHz, DMSO-*d6*) of **2d**

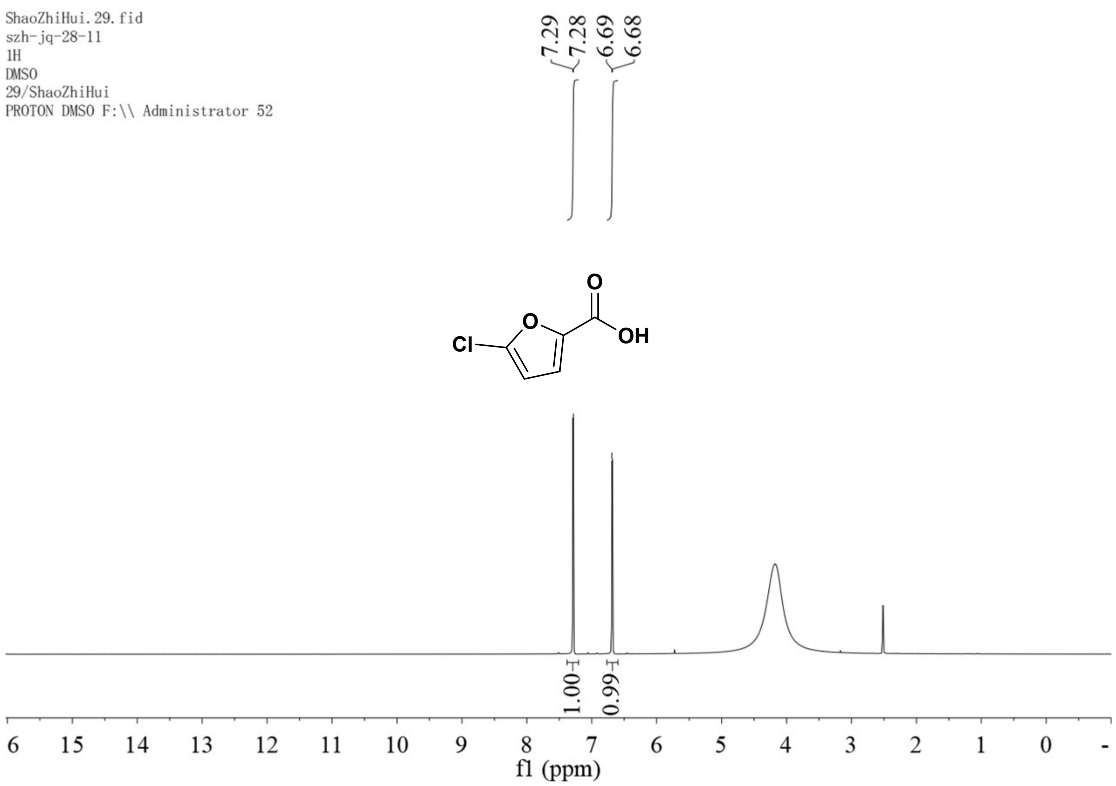

^{1}H NMR-spectrum (400 MHz, DMSO-*d*6) of **2e**

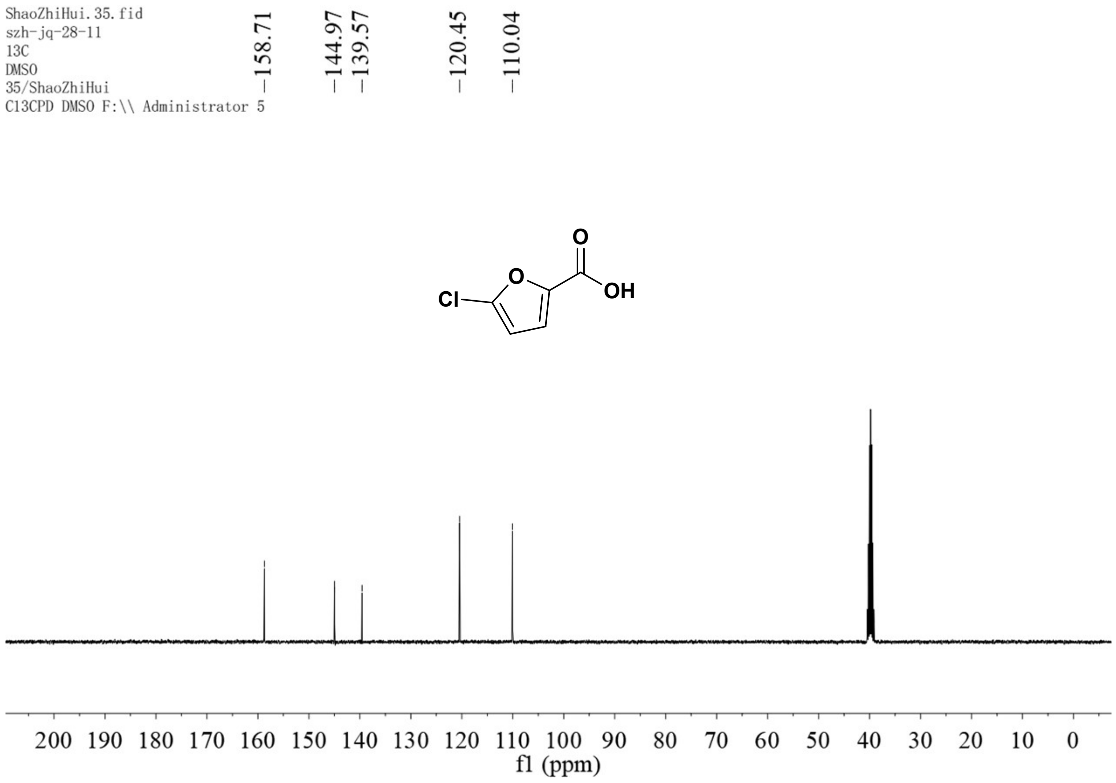

^{13}C NMR-spectrum (101 MHz, DMSO-*d*6) of **2e**

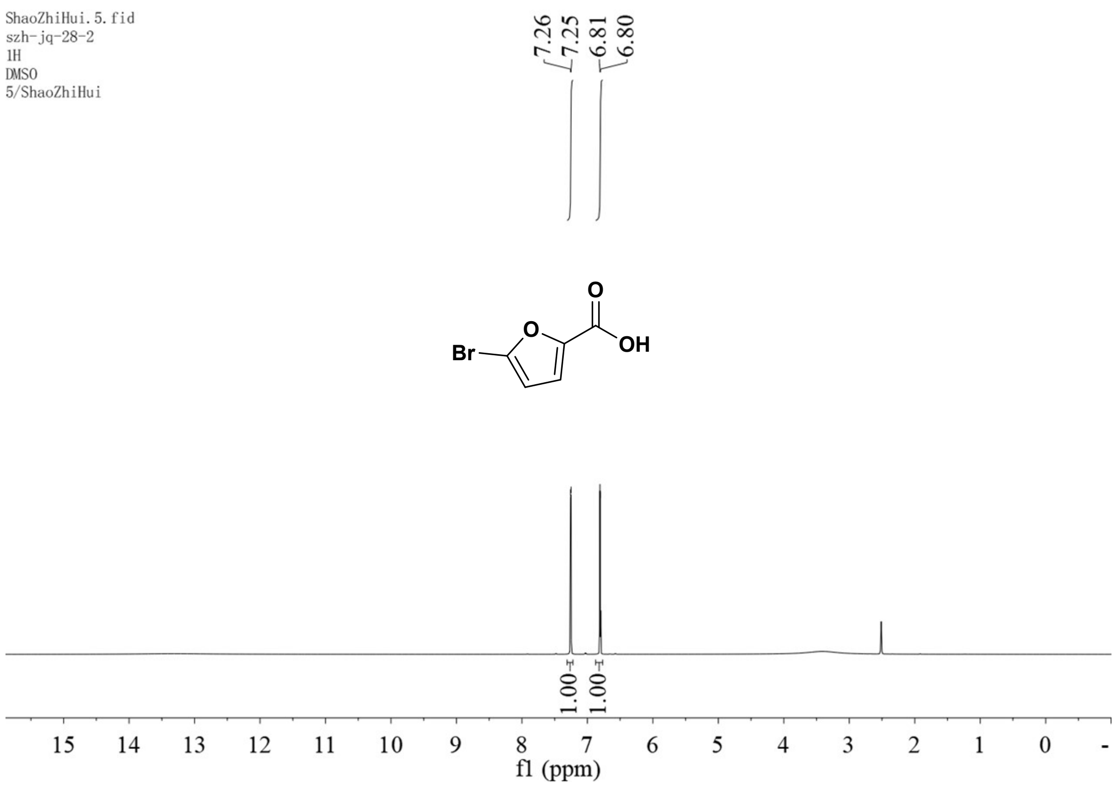

^{1}H NMR-spectrum (400 MHz, DMSO-*d*6) of **2f**

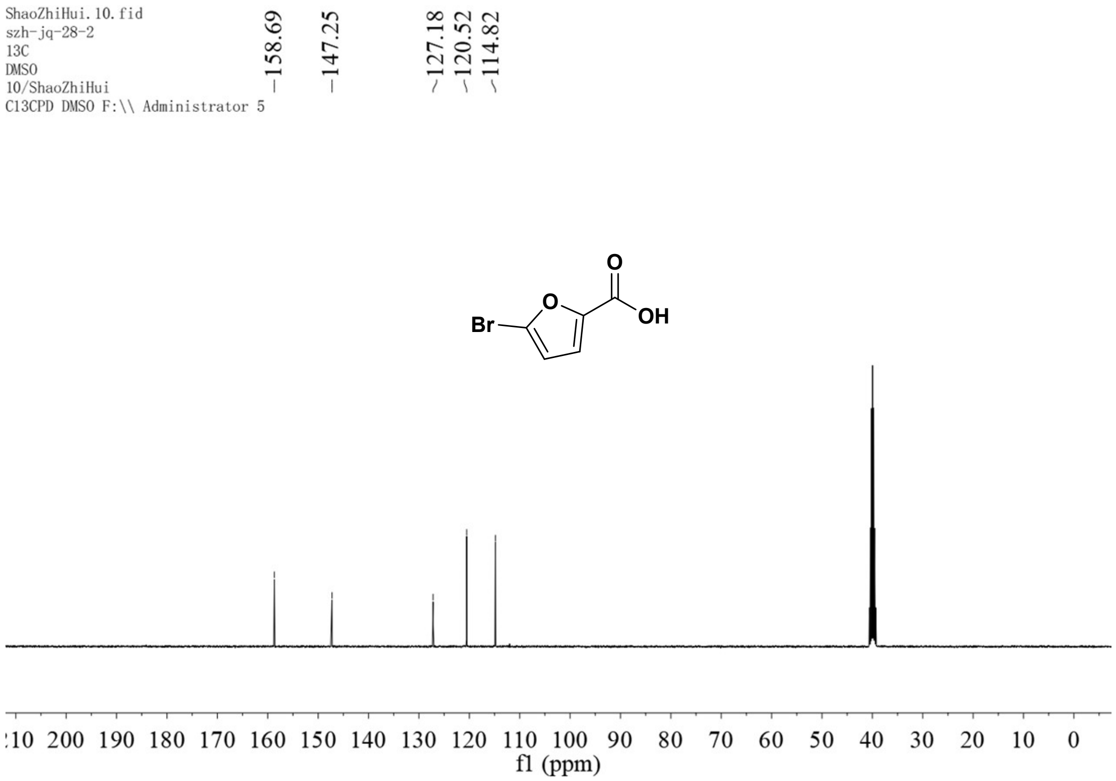

^{13}C NMR-spectrum (101 MHz, DMSO-*d*6) of **2f**

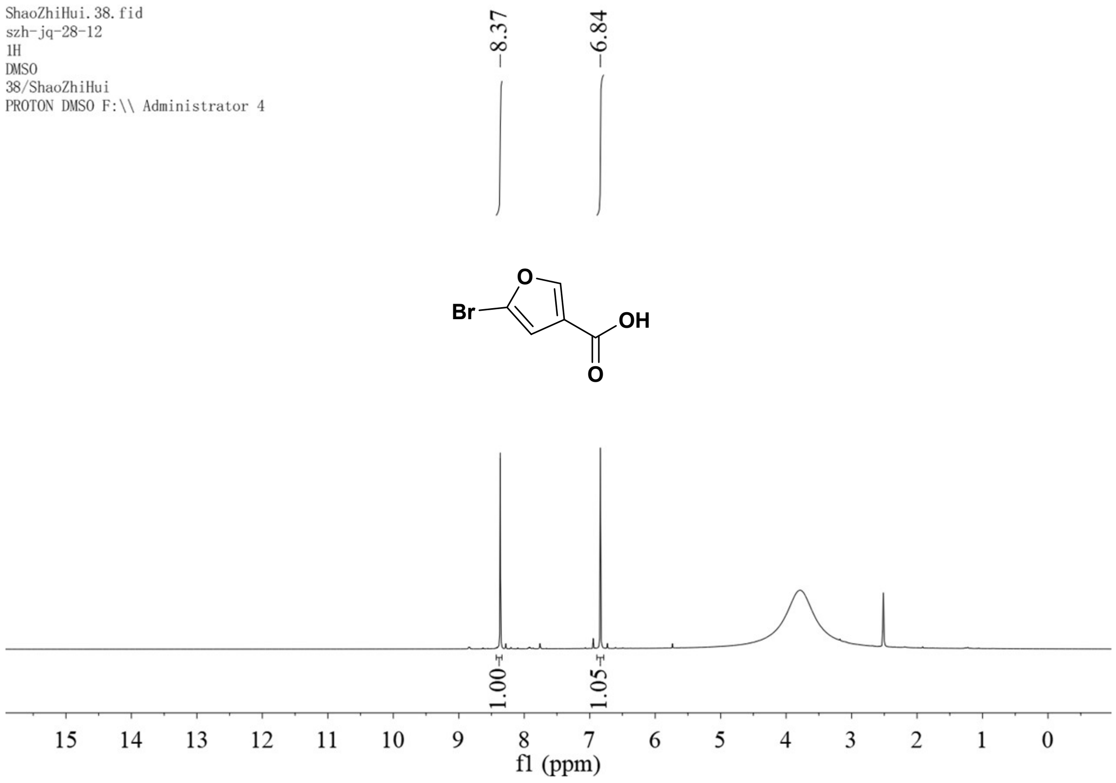

^{1}H NMR-spectrum (400 MHz, DMSO-*d6*) of **2g**

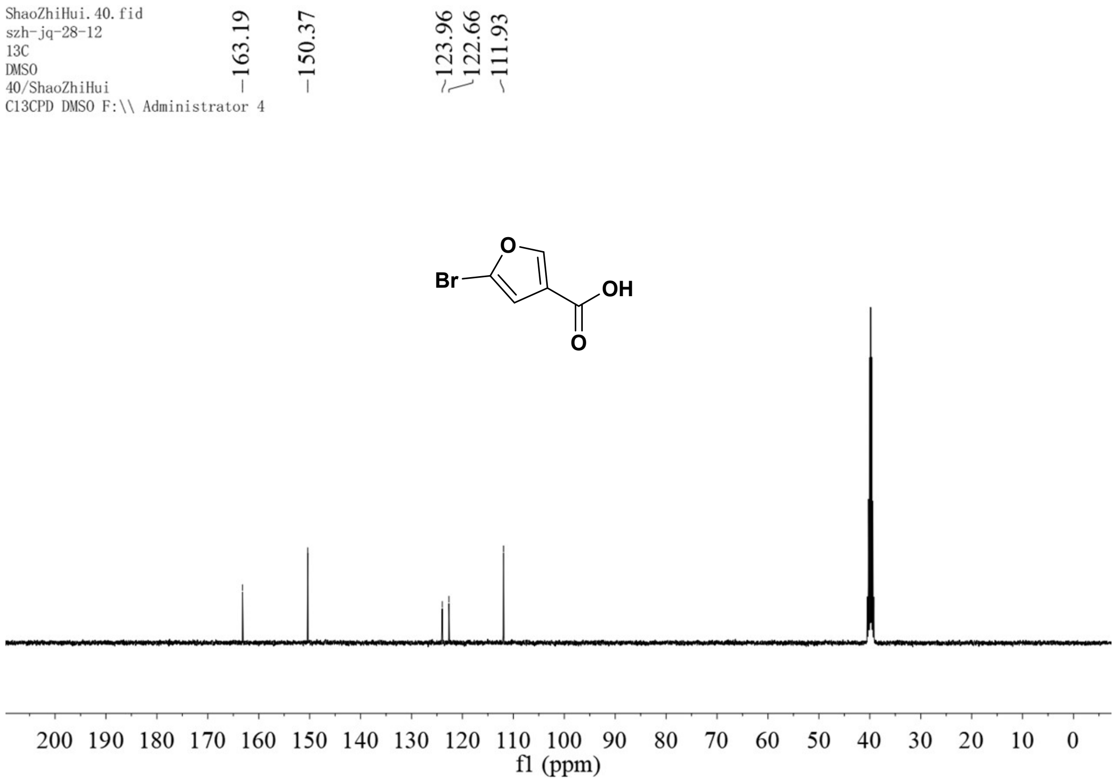

^{13}C NMR-spectrum (101 MHz, DMSO-*d6*) of **2g**

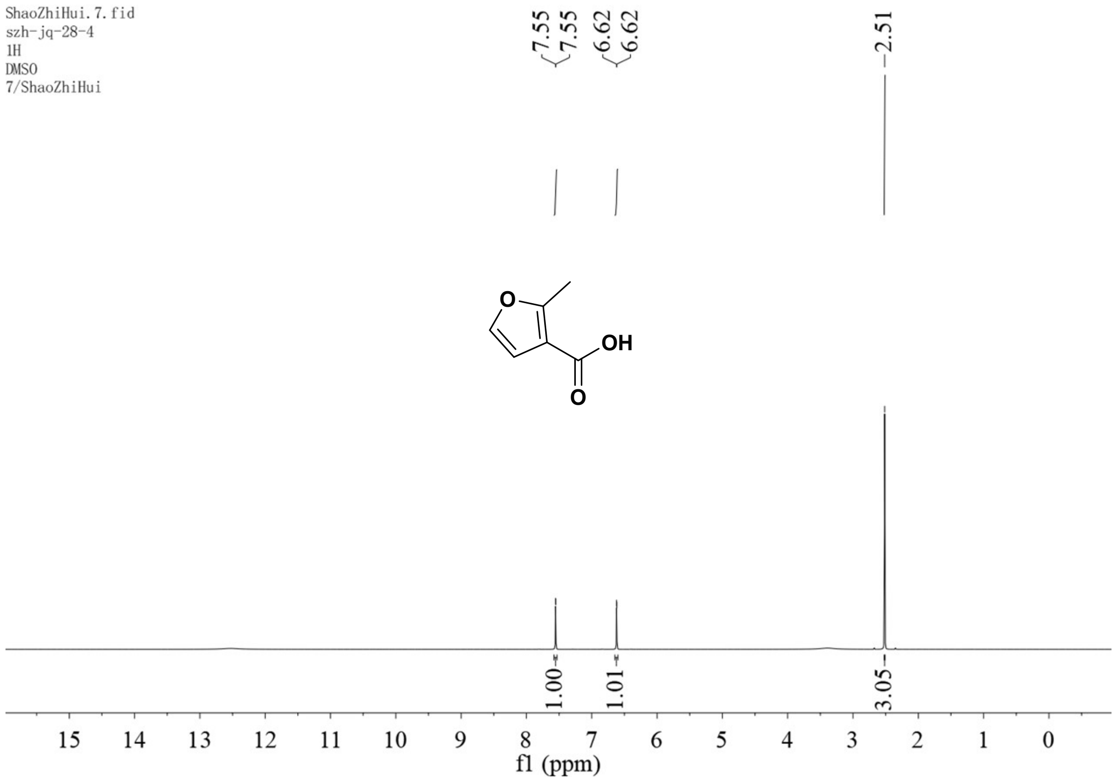

^{1}H NMR-spectrum (400 MHz, DMSO-*d6*) of **2h**

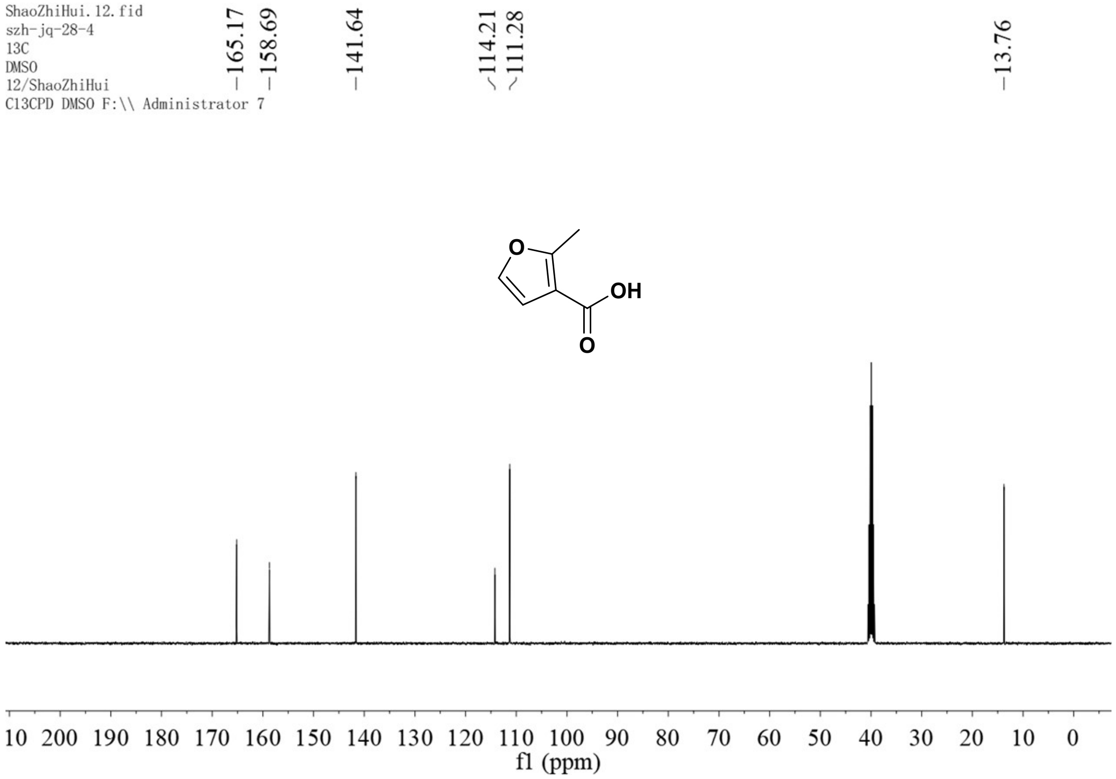

^{13}C NMR-spectrum (101 MHz, DMSO-*d6*) of **2h**

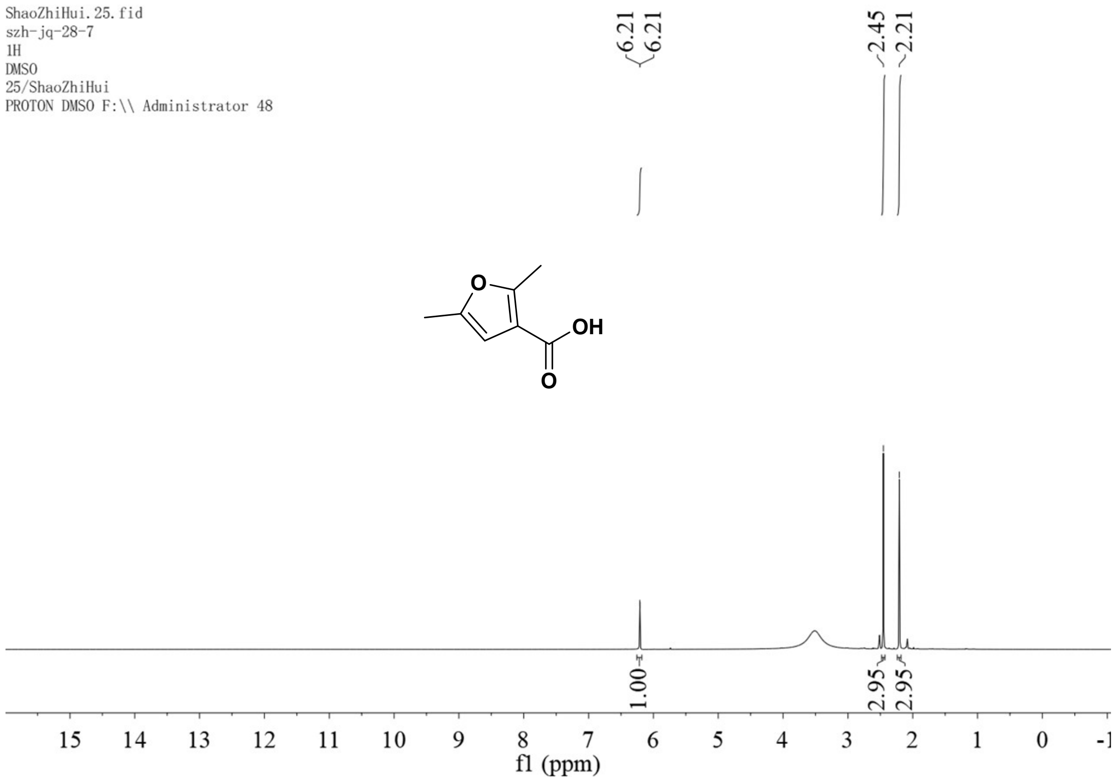

^{1}H NMR-spectrum (400 MHz, DMSO-*d*6) of **2i**

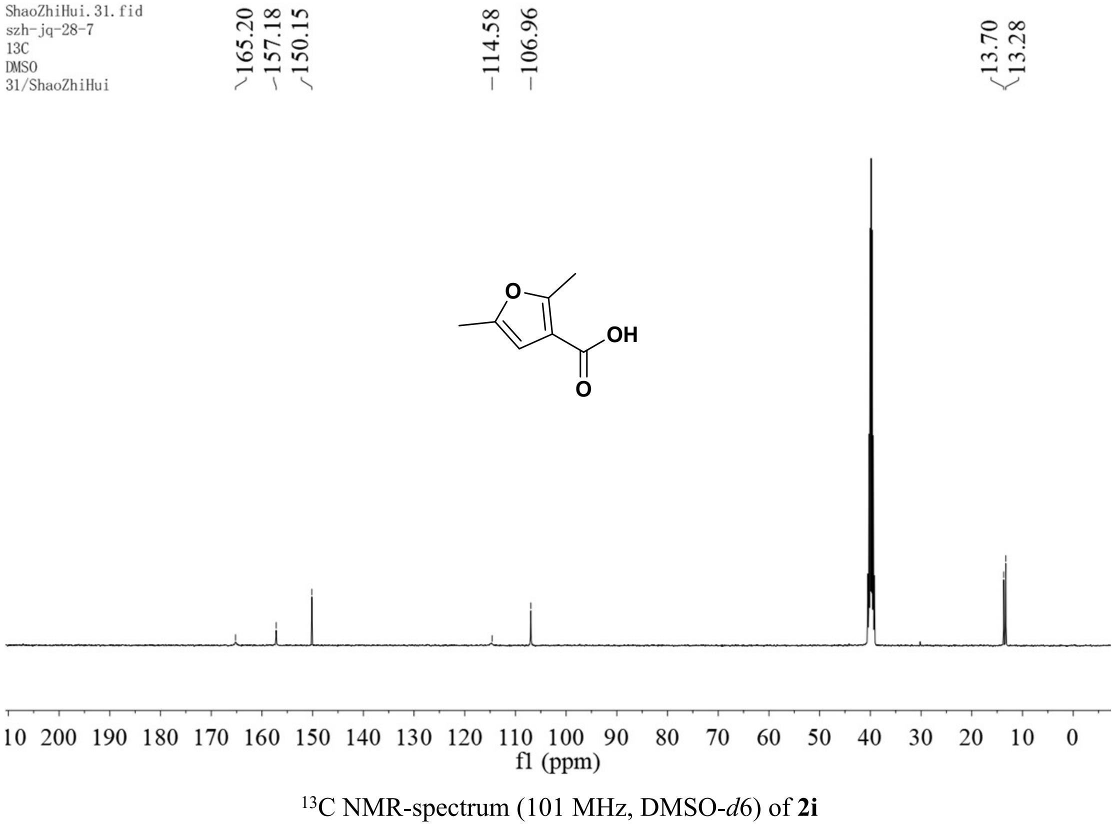

^{13}C NMR-spectrum (101 MHz, DMSO-*d*6) of **2i**

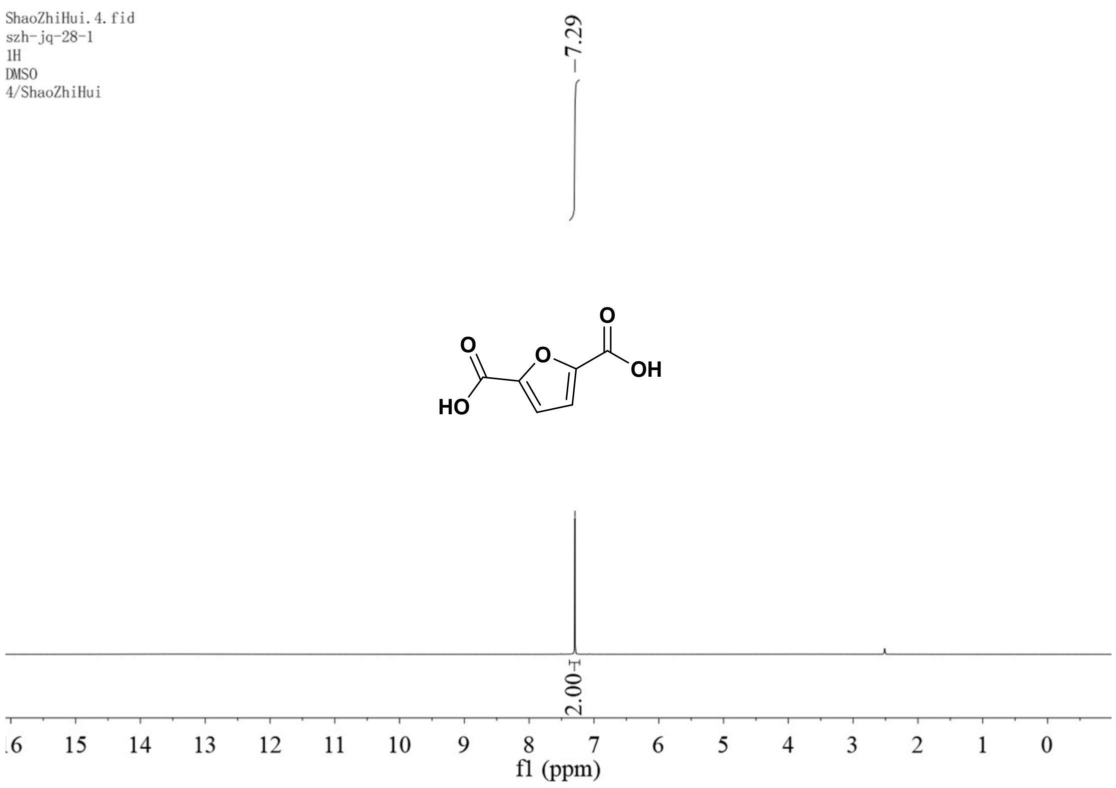

^{1}H NMR-spectrum (400 MHz, DMSO-*d6*) of **5a**

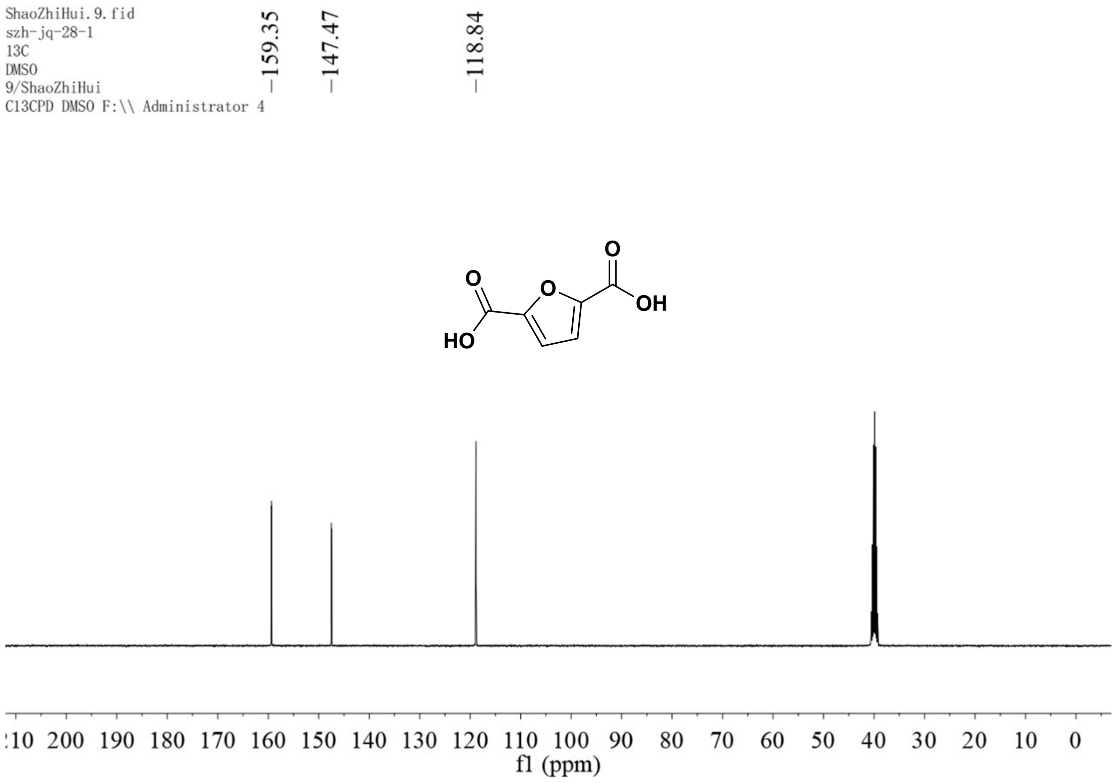

^{13}C NMR-spectrum (101 MHz, DMSO-*d6*) of **5a**

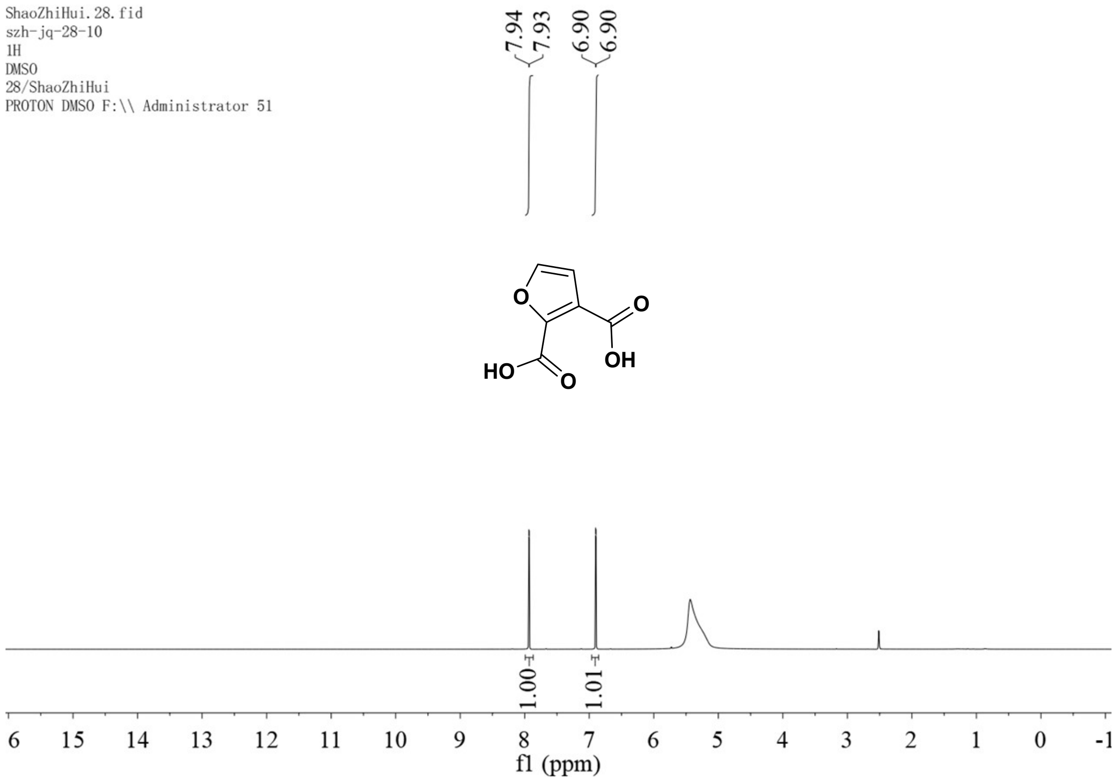

^{1}H NMR-spectrum (400 MHz, DMSO-*d6*) of **5b**

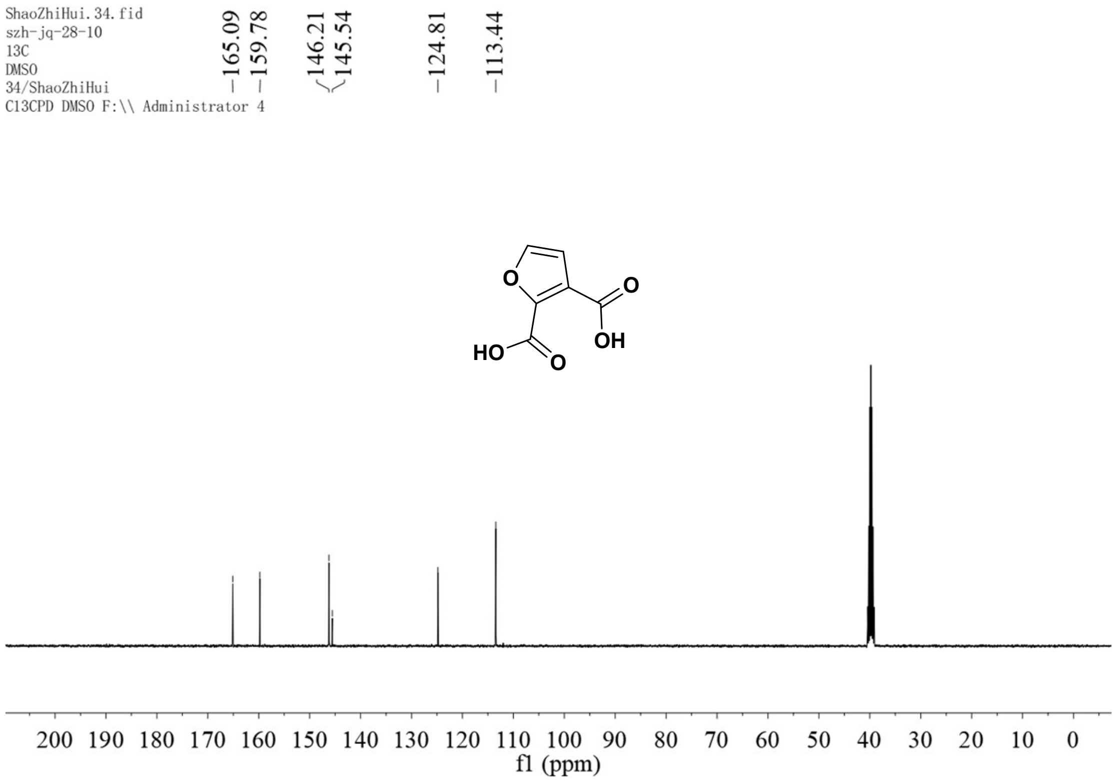

^{13}C NMR-spectrum (101 MHz, DMSO-*d6*) of **5b**

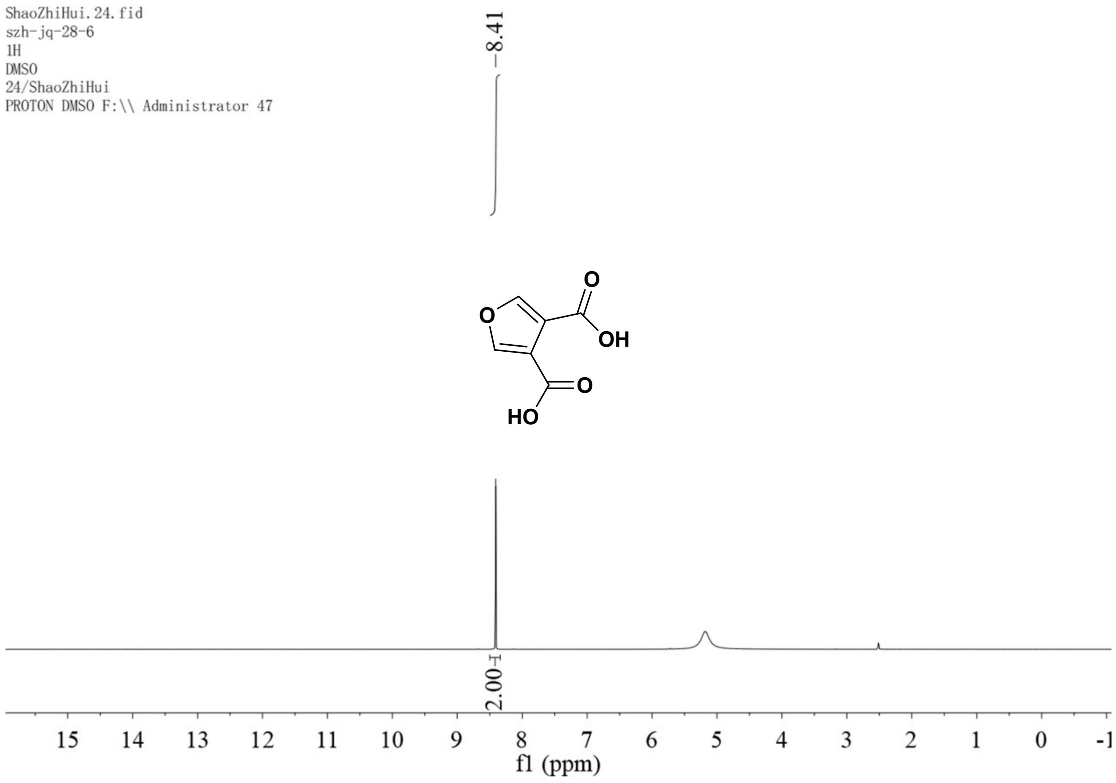

^{1}H NMR-spectrum (400 MHz, DMSO-*d*6) of **5c**

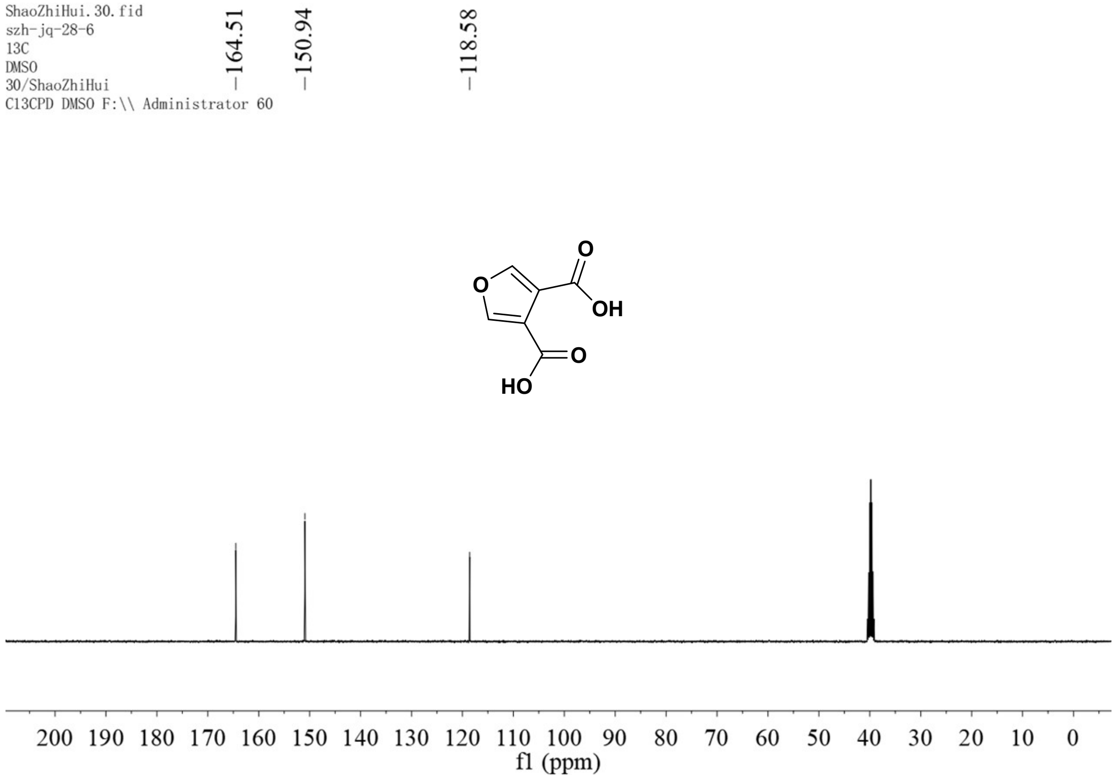

^{13}C NMR-spectrum (101 MHz, DMSO-*d*6) of **5c**
